# The Potential of Ferroptosis-Targeting Therapies for Alzheimer’s Disease: From Mechanism to Transcriptomic Analysis

**DOI:** 10.3389/fnagi.2021.745046

**Published:** 2021-12-20

**Authors:** Nad’a Majerníková, Wilfred F. A. den Dunnen, Amalia M. Dolga

**Affiliations:** ^1^Research School of Behavioural and Cognitive Neuroscience, University of Groningen, Groningen, Netherlands; ^2^Department of Pathology and Medical Biology, University Medical Centre Groningen, University of Groningen, Groningen, Netherlands; ^3^Department of Molecular Pharmacology, Groningen Research Institute of Pharmacy, University of Groningen, Groningen, Netherlands; ^4^Research Institute Brain and Cognition, Molecular Neuroscience and Aging Research (MOLAR), University Medical Centre Groningen, Groningen, Netherlands

**Keywords:** neurodegeneration, iron dysregulation, glutathione, lipid peroxidation, amyloid β

## Abstract

Alzheimer’s disease (AD), the most common form of dementia, currently affects 40–50 million people worldwide. Despite the extensive research into amyloid β (Aβ) deposition and tau protein hyperphosphorylation (p-tau), an effective treatment to stop or slow down the progression of neurodegeneration is missing. Emerging evidence suggests that ferroptosis, an iron-dependent and lipid peroxidation-driven type of programmed cell death, contributes to neurodegeneration in AD. Therefore, how to intervene against ferroptosis in the context of AD has become one of the questions addressed by studies aiming to develop novel therapeutic strategies. However, the underlying molecular mechanism of ferroptosis in AD, when ferroptosis occurs in the disease course, and which ferroptosis-related genes are differentially expressed in AD remains to be established. In this review, we summarize the current knowledge on cell mechanisms involved in ferroptosis, we discuss how these processes relate to AD, and we analyze which ferroptosis-related genes are differentially expressed in AD brain dependant on cell type, disease progression and gender. In addition, we point out the existing targets for therapeutic options to prevent ferroptosis in AD. Future studies should focus on developing new tools able to demonstrate where and when cells undergo ferroptosis in AD brain and build more translatable AD models for identifying anti-ferroptotic agents able to slow down neurodegeneration.

## Introduction

Alzheimer’s disease (AD) is the most prevalent age-related neurodegenerative disorder, affecting over 44 million people worldwide (Gaugler et al., 2016). In AD, formation of amyloid β (Aβ) plaques and neurofibrillary tangles (NFTs) are associated with progressive cortical and hippocampal neuronal dysfunction and death (Dugger and Dickson, 2017). Many cell death mechanisms have been studied in AD pathology. The aggregation of Aβ was linked with caspase-9 and caspase-3-dependant apoptosis in neurons (Obulesu and Lakshmi, 2014), autophagy deficiency (Li and Sun, 2017), necrosis (Tanaka et al., 2020) and microglia-dependant activation of inflammasome pathway (Heneka et al., 2018). Despite extensive research into main hallmarks and molecular pathways of cell death in AD, many degenerative processes cannot be explained by these mechanisms alone, resulting in failure of over 200 AD drugs trials aiming at these targets over the past decade (Yiannopoulou et al., 2019).

In addition to apoptosis and necrosis, ferroptosis, an iron dependent and lipid-peroxidation driven cell death (Dixon, 2017), seems to be associated with AD (Hambright et al., 2017). Ferroptosis, the process increasing with aging (Zhou et al., 2020), is morphologically, genetically, and biochemically different from other types of cell death (Dixon et al., 2012). Its hallmarks, such as increased iron levels and oxidative stress, have been long noted in the AD brain (Praticò et al., 2001; Praticò and Sung, 2004; Castellani et al., 2007; Derry et al., 2020). It has been shown that formation of Aβ plaques and NFTs is related to iron overload in AD models and post mortem tissue (Yamamoto et al., 2002; Peters et al., 2018). Moreover, iron levels positively correlate with cognitive decline in human subjects (Ayton et al., 2017), and glutathione peroxidase (GPx4, also known as GPX4), the critical regulator of ferroptosis, is protective in AD mice model (Yoo et al., 2010).

Human genome-wide association studies (GWAS) support these results by showing a relation between the risk of developing AD and *GPX4* polymorphism (Karch et al., 2016; da Rocha et al., 2018). Moreover, *PSEN1/2* mutations identified in Alzheimer patients affected the hypoxic response in mouse embryonic fibroblasts by regulating hypoxia inducible factor-1α (HIF-1α), a driver of vulnerability to ferroptosis in cancer (Kaufmann et al., 2013; Zou et al., 2019). These results suggest that higher risk of developing AD is associated with deregulation of ferroptosis-related proteins, and thus ferroptosis inhibitors may have a therapeutic potential in AD (Weiland et al., 2019). However, the underlying mechanism of ferroptosis in AD, and whether ferroptosis happens at the onset, during or as a consequence of AD remains to be established.

Our aim is to examine the potential of ferroptosis inhibition as a therapeutic strategy for AD. We will first recapitulate ferroptosis pathway and its relation to AD, identify which ferroptosis-related genes are differentially expressed in AD and lastly, discuss the therapeutic options to prevent ferroptosis in AD.

## Processes Involved in the Underlying Pathway of Ferroptosis

**FIGURE 1:**
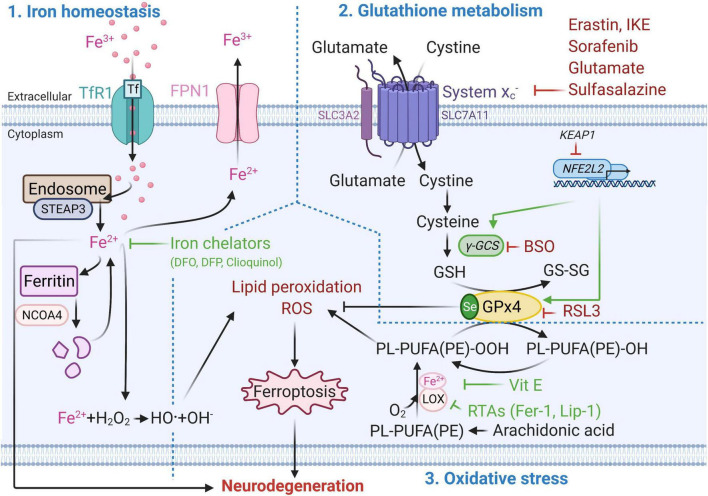
Molecular mechanisms of ferroptotic cell death. Metabolic pathways such as iron metabolism (left), cysteine and glutathione metabolism (top right), and polyunsaturated fatty acid metabolism (bottom right) play an essential role in the ferroptotic pathway. Well established ferroptosis inducers and inhibitors and their mode of action are depicted in red and green respectively. BSO, Buthionine sulphoximine; DFO, Deferoxamine; DFP, Deferiprone; Fe2 +, Ferrous iron; Fe3 +, Ferric iron; FPN1, Ferroportin; GPx4, Glutathione peroxidase 4; GSH, Glutathione (reduced glutathione form); GS-SG, Glutathione disulfide (oxidized glutathione form); Keap1, Kelch-like ECH-associated protein 1, LOX, Lipoxygenase; NCOA4, Nuclear receptor co-activator 4; NFE2L2, nuclear factor E2 related factor 2 encoding for Nrf2; PL-PUFA(PE)-OH, Polyunsaturated-fatty-acids (phosphatidylethanolamine)-reduced; PL-PUFA(PE)-OOH, Polyunsaturated-fatty-acid-containing-phospholipid hydroperoxides; ROS, Reactive oxygen species; RSL3, (1S,3R)-methyl-2-(2-chloroacetyl)-2,3,4,9-tetrahydro-1-[4-(methoxycarbonyl)phenyl]-1H-pyrido [3,4-b]indole-3-carboxylic acid; RTAs, Radical-trapping antioxidants; Se, Selenocysteine; STEAP3, Six-Transmembrane Epithelial Antigen Of Prostate 3; xCT subunit of system x_c_^–^, Glutamate/cystine antiporter system; TfR1, Transferrin 1 receptor; Vit E, Vitamin E; γ-GCS, Gamma-glutamylcysteine synthetase. This figure was created using Biorender.

Ferroptosis mechanism can be divided into three parts: (1) iron homeostasis, (2) glutathione (GSH) metabolism and (3) oxidative stress and lipid peroxidation (Figure 1). Disruption of one or more of these mechanisms can induce lipid peroxidation-driven ferroptotic cell death.

### Iron Homeostasis

Iron homeostasis plays a key role in ferroptosis (Yan and Zhang, 2020). Iron can enter the cell via transferrin 1 receptor (TfR1, also known as TFR1) and be reduced from ferric (Fe^3+^) to ferrous (Fe^2+^) form via metalloreductase STEAP3 in the endosome (Zhang et al., 2012). In this form, iron can be stored in ferritin, or exported from the cell via ferroportin (FPN1) (Chang, 2019). Ferritin degradation via the nuclear receptor coactivator 4 (NCOA4) contributes to ferroptosis by increasing the free intracellular iron levels (Hou et al., 2016). Excessive intracellular iron accumulation can lead to generation of reactive oxygen species (ROS) and oxidative stress via the Fenton reaction (Ward et al., 2014). Iron accumulation-induced ROS, such as superoxide anion (O_2_-•) and hydroxyl radical (•OH), possess an unpaired electron at their outer orbit which allows them to react with all cellular components including proteins, lipids and nucleic acid. This results in lipid peroxidation, oxidative damage to membranes and other lipid-containing molecules, and ultimately to cellular damage and ferroptotic cell death (Aprioku, 2013).

### Glutathione Metabolism

On the other hand, inhibition of glutamate/cystine antiporter (system x_c_^–^, xc-, with xCT as the functional subunit of system x_c_^–^) and depletion of GSH cause inactivation of GPx4, the critical antioxidant enzyme and regulator of ferroptosis (Seibt et al., 2019). This can lead to ferroptotic cell death through increased lipid peroxidation and accumulation of ROS (Wang et al., 2020). GPx4 reduces hydroperoxides of polyunsaturated-fatty-acid-containing-phospholipids (PL-PUFA(PE)-OOH) to polyunsaturated-fatty-acids (phosphatidylethanol amine)-reduced (PL-PUFA(PE)-OH) (Seibt et al., 2019). GPx4 uses GSH as a reducing substrate and converts it into oxidized form, also referred to as glutathione disulphide (GS-SG) (Cozza et al., 2017). Apart from nuclear factor erythroid 2-related factor 2 (Nrf2, coded by *NFE2L2* gene) (Habib et al., 2015), the xCT mRNA can be positively regulated by the activation of transcription factor 4 (ATF4) under oxidative stress (Sato et al., 2004), while its negative regulation by p53 results in cysteine deprivation and increased susceptibility to ferroptosis (Jiang et al., 2015).

### Oxidative Stress and Lipid Peroxidation

Oxidative stress occurs due to the imbalance between generation of free radicals and the ability to neutralize or eliminate them through antioxidants (Birben et al., 2012). One of the main drivers of ferroptosis is ROS-mediated lipid peroxidation, which can result in oxidative stress (Kuang et al., 2020). Inhibition of GPx4 and decrease in GSH levels lead to activation of 12/15-lipoxygenase (12/15-LOX, which is the protein product of the ALOX15 gene). The association of Fe^2+^ with lipoxygenases (LOX, a dioxygenase containing non-heme iron) can lead to oxygenation of polyunsaturated-fatty-acids (PUFA), such as arachidonic acid present in phospholipids, and trigger lipid peroxidation-induced ferroptosis (Kagan et al., 2017). The LOX nomenclature is defined by the specific site of their oxygenation product: in humans there are six LOX isoforms 15-LOX-1, 15-LOX-2, 12-LOX-1, 12-LOX-2, E3-LOX, and 5-LOX, of which 12/15-LOX (15-LOX) are the most abundant. 12/15-LOX are considered as one of the key regulators of ferroptotic cell death (Yang et al., 2016; Kagan et al., 2017). Although, this has been supported by the findings that pharmacological inhibition of 15-LOX-1 exerts a cytoprotective effect (Seiler et al., 2008; Eleftheriadis et al., 2016), some off-target effects of lipoxygenase inhibitors have also been reported (Shah et al., 2018).

In addition to iron accumulation-induced generation of ROS, mitochondria also contribute to ROS production. Electrons leak from complex I and III of the electron transport chain (ETC) located on the inner membrane of mitochondria (Zhao et al., 2019). This can result in the formation of ROS such as O_2_-• and hydrogen peroxides (H_2_O_2_), and potentially can lead to loss of mitochondrial membrane potential (ΔΨm) (Gao et al., 2019). Reduced ΔΨm was associated with ferroptosis and involves different regulatory mechanisms than apoptosis (Kuang et al., 2020). GSH depletion-induced activation of 12/15-LOX can increase cytosolic Ca^2+^ via both (1) the import from the extracellular compartment and (2) release from mitochondria and endoplasmic reticulum (Maher et al., 2018). Decrease in GSH levels can also lead to dysregulation of Ca^2+^ transport in and out of mitochondria by voltage dependant anion channels (VDAC) and mitochondrial Ca^2+^ uniporter (MCU) (Zorov et al., 2014; DeHart et al., 2018). This results in mitochondrial Ca^2+^ overload and collapse of the mitochondrial function which activates Ca^2+^-dependant proteases (Zorov et al., 2014; DeHart et al., 2018; Marmolejo-Garza and Dolga, 2021). Consequently, ROS-induced transactivation of BH3 interacting-domain death agonist (BID) to mitochondria and Ca^2+^overload-induced translocation of apoptosis-inducing factor (AIF) from mitochondria to the nucleus causes the cell to die (Neitemeier et al., 2017). This caspase-independent process is accompanied by mitochondrial fragmentation and enlarged cristae (Dixon et al., 2012). The rescue of mitochondria (Jelinek et al., 2018), decrease of mitochondria-associated endoplasmic reticulum membranes (MAMs) interaction (Guo et al., 2019) and small conductance calcium-activated potassium (K_Ca_2/SK) channel activation have the potential to protect from ferroptotic cell death (Krabbendam et al., 2020).

## Contributions of Ferroptosis to Alzheimer’s Disease

### Iron Homeostasis

Advanced age is associated with iron dysregulation affecting most of our organs (Xu et al., 2012; Picca et al., 2019). Many studies show that iron dysregulation can also contribute to AD pathology (Bush, 2013; Nuñez and Chana-Cuevas, 2018). With aging, iron deposits in the brain (Acosta-Cabronero et al., 2016), which can increase the formation of Aβ plaques (Becerril-Ortega et al., 2014) and tau hyperphosphorylation in AD transgenic mouse brain (Guo et al., 2013). Imaging and histological experiments support this by showing increased iron deposition in AD-specific brain regions (Altamura and Muckenthaler, 2009; Bush, 2013; Apostolakis and Kypraiou, 2017; Lee and Lee, 2019). Magnetic resonance imaging (MRI) studies revealed increased iron levels in the putamen, pulvinar thalamus, red nucleus, hippocampus, and temporal cortex of AD patients (Langkammer et al., 2014). Later, quantitative susceptibility mapping showed higher iron levels in caudate and putamen nucleus of AD patients than in controls. Interestingly, the increased iron level in the left caudate nucleus correlated with the degree of cognitive impairment (Du et al., 2018). Finally, higher iron levels in the frontal cortex were associated with AD severity (Bulk et al., 2018b). This evidence suggested that iron contributes to AD pathology and presented an important avenue for therapy development (Masaldan et al., 2019).

### Glutathione Metabolism

Ferroptosis can be induced by compounds interfering with system x_c_^–^, such as erastin, which induces cysteine deprivation, GSH depletion, endoplasmic reticulum stress, and cell death (Dixon et al., 2012, 2014; Sato et al., 2018). System x_*c*_^–^ can also be inhibited by adding small concentrations of sorafenib (Lachaier et al., 2014), glutamate (Jiang et al., 2020) and sulfasalazine (Yu et al., 2019) to the extracellular compartment. Inhibition of gamma-glutamylcysteine synthetase (γ*-*GCS) by buthionine sulphoximine (BSO) results in GSH depletion and can lead to ferroptosis (Griffith, 1982). Irreversible and direct inhibition of GPx4 by the (1S,3R)-RSL3 (RSL3), causes the production of polyunsaturated-fatty-acid-containing-phospholipid hydroperoxides, which leads to lipid peroxidation and ferroptotic cell death (Liang et al., 2019). In addition to pharmacological compounds, genetic modifications targeting regulators of the system x_c_^–^ can induce ferroptosis. The *Gpx4*BI-KO mouse was generated by a conditional deletion of *Gpx4* in forebrain neurons by administration of tamoxifen. In this mouse model, 75–85% decrease of *Gpx4* was shown to induce hippocampal neuronal loss, lipid peroxidation, neuroinflammation and spatial learning deficits (Hambright et al., 2017). Similarly, the knockout of *Gpx1*, facilitated memory impairment induced by β-Amyloid in mice (Joo et al., 2020). The Western blot analysis of AD post mortem brain tissue revealed enhanced expression of the light-chain subunit of the xCT (Ashraf et al., 2020). These results suggest that impaired GSH metabolism might play a role in ferroptosis during AD pathology (Ashraf et al., 2020).

### Oxidative Stress and Lipid Peroxidation

The brain is the most vulnerable organ to oxidative stress. It represents only 2% of the body but uses 20% of the total oxygen supply (Sokoloff, 1999). Oxidative stress plays a key role in AD pathology by initiating the generation and enhancing of both Aβ plaques and hyperphosphorylation of Tau (p-Tau) (Huang et al., 2016; Nassireslami et al., 2016). Oxidative stress can be enhanced in AD via metal accumulation. In addition to iron, the Aβ precursor protein (APP) has a high affinity to binding zinc and copper at the N terminal metal-binding sites (Barnham et al., 2003). Additionally, high concentrations of these metals were also found in Aβ plaques in mouse and human brain (Plascencia-Villa et al., 2016; James et al., 2017). As copper is the potent mediator of •OH, and the binding of zinc leads to production of toxic Aβ and further uncontrolled zinc release, these metals can contribute to the increase of oxidative stress in AD (Strozyk et al., 2009). Post mortem tissue from AD patients shows higher levels of oxidized bases in the frontal, parietal and temporal lobes compared to control subjects (Wang et al., 2005), which correlates with imbalanced levels of copper, zinc and iron (Deibel et al., 1996). Other studies have shown higher level of lipid peroxidation, in diseased regions of AD brain compared to controls (Montine et al., 1998; Lovell et al., 2001; Bradley-Whitman and Lovell, 2015). These results support that oxidative stress might be an important factor contributing to the development and progression of AD (Zhao and Zhao, 2013).

## Differential Expression of Ferroptosis-Related Genes in Alzheimer’s Disease

Many AD differentially expressed genes (DEGs) have been identified in animal and human studies. Using available RNAseq datasets of AD mouse models, AD patients and age-matched controls, we analyzed which of the 44 ferroptosis-related genes are differentially expressed in AD (Supplementary Table 1). To this end, we analyzed the expression of ferroptosis-related genes in one mouse [Alzmap (Chen et al., 2020)] and three human datasets of AD-DEGs [scREAD (Mathys et al., 2019), ACTA (Gerrits et al., 2021), AMPA-AD (Wan et al., 2020)]. All four datasets were available to the public and compared the gene expression between cell types, stages of disease progression and gender.

**FIGURE 2:**
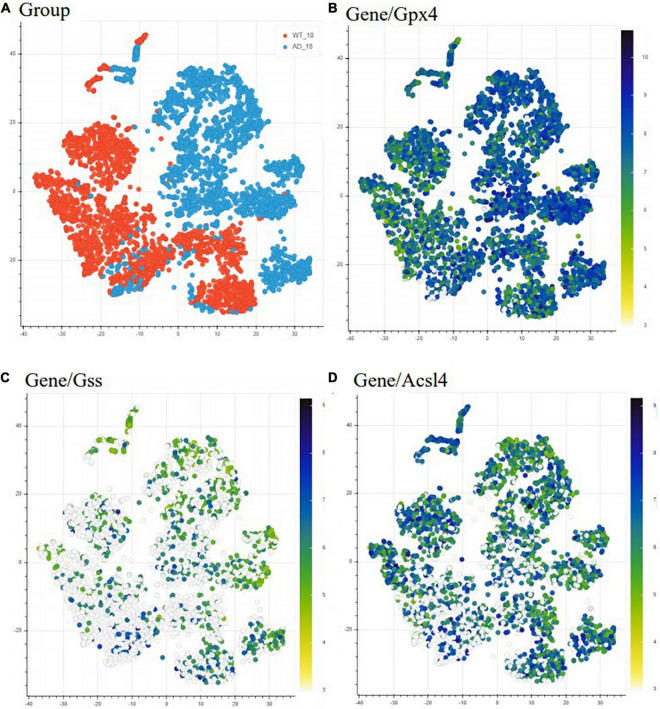
Differential expression of ferroptosis related genes in AD mice model compared to WT mice. Heatmap representing the difference in expression of ferroptosis-related genes between 18 months old WT (orange) and AD mice (light blue) **(A)**. The heatmap depicts gene expression from low/white to high/dark blue. Each point indicates one spatial transcriptomic spot defining one tissue domain on the slide. Glutathione peroxidase 4 (Gpx4) is upregulated with pathology **(B)**, while glutathione synthase (Gss) **(C)** and acyl-CoA synthetase long-chain family member (Acsl4) **(D)** are downregulated with the pathology. This data is freely accessible online, Alzmap (Chen et al., 2020).

We first used the Alzmap gene retrieving function to make a qualitative assessment of the expression of three representative ferroptosis-related genes. We included (i) *Gpx4*, as it can suppress phospholipid peroxidation, an important process during ferroptosis, (ii) *Gss*, as it can facilitate the production of GSH, and (iii) *Acsl4* for its role in supporting the incorporation of long PUFAs into lipid membranes, a process associated with ferroptosis (Figure 2). We choose t-distributed stochastic neighbor embedding (TSNE) statistical method to visualize the representative genes in a high-dimensional dataset (Figure 2). However, Alzmap website offers other modes of analysis and visualization tools such as the principal component analysis (PCA) and uniform manifold approximation and projections for dimension reduction (UMAP). The distribution and visualization of the chosen genes might render different output since these methods of visualization and reduction tools are based on specific clustering algorithms, i.e., unsupervised linear dimensionality reduction and data visualization technique for very high dimensional data for PCA, while t-SNE is based on a non-linear statistical method, calculating the similarity probability score in a low dimensional space. Therefore, visualization of genes could appear to render various outcomes. The alterations observed in the ferroptosis-related genes generated by Alzmap are purely based on a qualitative assessment. These data can be freely accessible on the https://alzmap.org/website.

In the Alzmap study, one left and one right hemisphere was collected for each experimental group and analyzed according to the spatial transcriptomic manual (Stockholm, Sweden) (Ståhl et al., 2016) using Fiji groovy script package (Chen et al., 2020). Our analysis revealed *Gpx4* upregulation and *Gss* and *Acsl4* downregulation in *App^NL-G-F^* knock-in AD mice compared to WT mice. Although this analysis shows that these ferroptosis-related genes are differentially expressed in *App^NL-G-F^* knock-in AD mice, it is known that downregulation of *Gpx4* and upregulation of *Acsl4* can induce ferroptosis (Dixon et al., 2012). Our observation from the TSNE analysis can be explained by cells trying to increase resistance against ferroptosis by increasing the generation of antioxidants (from the observation of increased *Gpx4*) and depleting the substrates for lipid peroxidation (as *Acsl4* gene expression was found decreased) (Stockwell et al., 2017).

In the second study containing the scREAD dataset (Mathys et al., 2019), 48 participants were divided into early and late stage groups based on nine clinical pathological traits. Data was acquired by single-nucleus RNA sequencing (snRNAseq)-based differential expression analysis and assessed by Wilcoxon rank-sum test and false discovery rate (FDR) multiple-testing correction (Mathys et al., 2019). Our analysis revealed that ferroptosis-related genes in excitatory neurons from human brains are mostly downregulated at an early clinical stage of AD, while they are upregulated at a later clinical stage of the disease relative to early stage (Table 1). The same was observed with inhibitory neurons, astrocytes and glia cells. For instance, genes which are important for ferroptosis resistance [e.g., *ACSL3*, ferritin heavy chain (*FTH1*), *GPX4*, *GSS* and voltage-dependent anion channel 2 and 3 (*VDAC2/3*)] are downregulated in an early stage of AD pathology but upregulated at later AD stage. This could imply that ferroptosis already happens at early stages of the diseases. The shift from downregulation to upregulation at later stages can be explained by cells trying to compensate and rescue the ferroptotic cell death by increasing the expression of antioxidant proteins and enzymes. Furthermore, the observation that neurons show a higher number of ferroptosis DEGs in AD than astrocytes and oligodendrocytes suggests that ferroptosis affects neurons and glia cells differently (Kim et al., 2021). Although it seems from this dataset that ferroptosis gene expression changes primarily in neurons, it might be because glia cells were not primarily sorted out in this study. Therefore, next we analyzed a dataset that specifically looked at glia cells.

**TABLE 1:** Log2-fold change of ferroptosis-related DEGs related to AD.

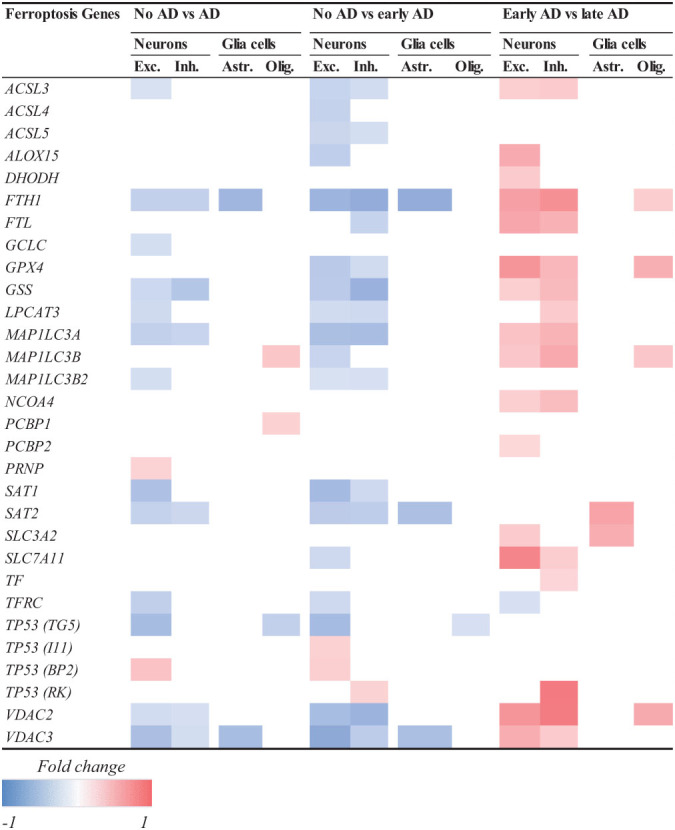

*Decreased (blue) and increased (red) expression of ferroptosis-related genes in neurons (Exc, Excitatory and Inh, Inhibitory) and glia cells (Ast, Astrocytes and Olig, Oligodendrocytes) in AD brain. White space corresponds to unchanged gene expression. Participants were divided into early and late stage groups based on 9 clinico-pathological traits. Early AD is associated with decrease and late AD with increase in ferroptosis-related gene expression.*

*bACSL3, Long-chain-fatty-acid-CoA ligase 3; ACSL4, Long-chain-fatty-acid-CoA ligase 4; ACSL5, Long-chain-fatty-acid-CoA ligase 5; ALOX15, coding for arachidonate 15-lipoxygenase/15-lipoxygenase-1; DHODH, Dihydroorotate dehydrogenase; FTH1, Ferritin heavy chain; FTL, Ferritin light chain; GCLC, Glutamate-cysteine ligase catalytic subunit; GPx4, Glutathione peroxidase 4; GSS, Glutathione synthetase; LPCAT3, Lysophosphatidylcholine acyltransferase 3; MAP1LC3A, Microtubule associated protein 1 light chain 3 Alpha; MAP1LC3B, Microtubule associated protein 1 light chain 3 Beta; MAP1LC3B2, Microtubule associated protein 1 light chain 3 Beta 2; NCOA4, Nuclear receptor coactivator 4; PCBP1, Poly(rC)-binding protein 1; PCBP2, Poly(rC)-binding protein 2; PRNP, prion protein; SAT1, Spermidine/spermine N1-acetyltransferase 1; SAT2, Spermidine/spermine N1-acetyltransferase 2; SLC11A2, Solute carrier family 11 member 2; TF, Transferrin; TFRC, Transferrin receptor; TP53BP2, Tumor protein p53 binding protein, 2; TP53I11, TP53 inducible protein; TP53RK, TP53 regulating kinase; TP53TG5, Tumor protein 53 target 5; VDAC2, Voltage-dependent anion channel 2; VDAC3, Voltage-dependent anion channel 3.*

*The criteria to determine if the change of the gene was significant included the false discovery rate (FDR)-corrected p < 0.01 in a two-sided Wilcoxon-rank sum test, absolute log_2_ > 0.25, and FDR-corrected P < 0.05 in a Poisson mixed model. Data was analyzed based on Mathys et al. (2019).*

To further investigate how ferroptosis could affect glia cells in AD, we looked at the difference in expression of ferroptosis-related genes in microglia between control and AD brains containing only amyloid-β plaques in the occipital cortex (OC) and both amyloid-β and tau pathology in the occipitotemporal cortex (OTC) (Gerrits et al., 2021). In this study, the differential expression analysis was performed using a logistic regression and adjusted *p*-value below 0.05 was used to determine the significance (Gerrits et al., 2021). Microglia belonging to different subclusters (homeostatic, Aβ-related = AD1 and tau-related = AD2) showed changes in the expression of ferroptosis-related genes between AD and control subjects (Table 2). Microglia affected by Aβ pathology alone, or the combination of Aβ and tau pathology showed more DEGs than cells in the homeostatic subcluster. Microglia in the Aβ-related subcluster showed increase in the expression of ferroptosis-related genes, while microglia in tau pathology-related subcluster showed decrease in the expression of these genes. As the presence of tau pathology in OC is typical for later stages of the diseases, these results could suggest that there seem to be a difference between the expression of ferroptosis-related genes between early and late stages of AD. However, whether glia cells die via ferroptotic cell death at later stages of AD should be investigated further.

**TABLE 2:** Log-fold change of ferroptosis-related DEGs in glia cells in AD.

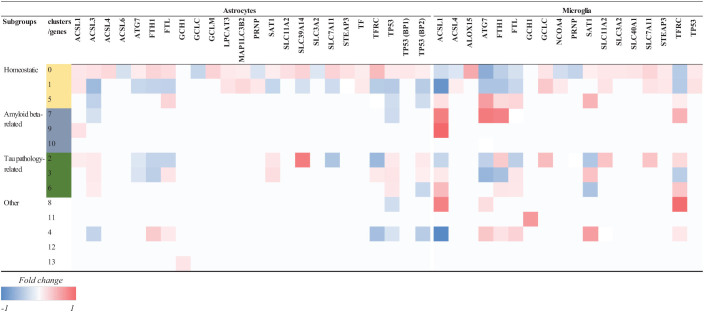

*Data in this table represent the Log-fold change per gene per subcluster. Decreased (blue) and increased (red) expression of ferroptosis-related genes in microglia nuclei isolated from CTR and AD brain tissues. Microglia were clustered into 13 subclusters, that were categorized as follows: 1. homeostatic, 2. Aβ-plaque associated (-AD1) and 3. Tau-associated (AD2), and other subclusters were related to pro-inflammatory responses, cellular stress and proliferation. White space corresponds to unchanged gene expression. ACSL, Long-chain-fatty-acid–CoA ligase; ALOX15, coding for Arachidonate 15-lipoxygenase/15-lipoxygenase-1; ATG, Autophagy related gene; FTH1, Ferritin heavy chain; FTL, Ferritin light chain; GCH1, Guanosine triphosphate cyclohydrolase-1; GCLC, Glutamate-cysteine ligase catalytic subunit; HMOX1, Heme oxygenase 1; NCOA4, Nuclear receptor coactivator 4; SAT1, Spermidine/spermine N1-acetyltransferase; SLC, Solute carrier family; STEAP3, STEAP3 Metalloreductase, TFRC, Transferrin receptor; TP53, tumor protein 53. The differential expression analysis was performed using a logistic regression from which we included ferroptosis-related genes with an adjusted p-value < 0.05. Differential gene expression results were extracted from supplementary table 2 from Gerrits et al. (2021).*

*ACSL, Long-chain-fatty-acid—CoA ligase; ALOX15, coding for Arachidonate 15-lipoxygenase/15-lipoxygenase-1; ATG, Autophagy related gene; FTH1, Ferritin heavy chain; FTL, Ferritin light chain; GCH1, Guanosine triphosphate cyclohydrolase-1; GCLC, Glutamate-cysteine ligase catalytic subunit; GCLM, Glutamate-cysteine ligase modifier subunit; LPCAT3, Lysophosphatidylcholine acyltransferase 3; MAP1LC3B2, Microtubule associated protein 3 light chain 2 Beta; NCOA4, Nuclear receptor coactivator 4; PRNP, Prion protein; SAT1, Spermidine/spermine N1-acetyltransferase; SLC, Solute carrier family; STEAP3, STEAP3 Metalloreductase, TF, Transferrin; TFRC, Transferrin receptor; TP53, tumor protein 53.*

*The differential expression of genes was determined using a ‘chisq.test’ function in R and ‘anova_test’ function from the rstatix package (Moran’s I test, q-value < 0.05). Data was analyzed based on Gerrits et al. (2021).*

Previous analysis of the whole brain human DEGs in AD revealed more AD-DEGs in women than men (Wan et al., 2020). To see whether this is also specifically true for ferroptosis-related genes, we analyzed the 44 ferroptosis-related genes in the AMPA-AD dataset where AD-DEGs were compared between genders (Table 3). The sample size included 478 AD (female: 318, male: 160) and 300 control (female: 148, male: 152) cases on which sex-stratified meta-analysis (Wan et al., 2020). Our analysis revealed three downregulated genes in both men and women while only *GSS* was downregulated in both. Only one gene, Cytochrome B-245 Beta Chain (*CYBB*), was upregulated in men while eleven genes were upregulated in women (Table 3). The analysis of the dataset available in this study indicates that like AD-DEGs, ferroptosis-related genes seem to be more differentially expressed in women than men. Finally, nine of the 44 ferroptosis-related genes were not differentially expressed in any of the analyzed datasets (Supplementary Table 1).

**TABLE 3:** Comparison of ferroptosis-related DEGs in AD between genders.

	Men	Women
Downregulated	*GSS, SLC11A2, TFRC*	*GSS, MAP1LC3A, VDAC3*
Upregulated	*CYBB*	*ACSL1, ALOX15B, FTL, HMOX1, NCOA4, SLC7A11, STEAP3, TF, TP53BP2, TP53I3, TP53RK*

*ACSL1, Long-chain-fatty-acid-CoA ligase 1; ALOX15, Arachidonate 15-lipoxygenase/15-lipoxygenase-1; CYBB, Cytochrome B-245 Beta chain; FTL, Ferritin light chain; GSS, Glutathione synthetase; HMOX1, Heme oxygenase 1; MAP1LC3A, Microtubule associated protein 1 Light chain 3 Alpha; NCOA4, Nuclear receptor coactivator 4; SLC11A2, Solute carrier family 11 member 2; SLC7A11, Solute carrier family 7 member 11; STEAP3, STEAP3 Metalloreductase; TF, Transferrin; TFRC, Transferrin receptor; TP53BP2, Tumor protein p53 binding protein, 2; TP53I3, TP53 inducible protein; TP53RK, TP53 regulating kinase; VDAC3, Voltage-dependent anion channel 3.*

*The differentially expressed genes were determined as those with FDR P < 0.05 using weighted fixed/mixed effect linear models using the ‘voom-limma’ R package. Data was analyzed based on Wan et al. (2020).*

## Inhibition of Ferroptosis to Treat Alzheimer’s Disease

An increasing amount of literature suggests that anti-ferroptotic therapies may be efficient in AD (Ashraf et al., 2020; Li et al., 2020; Table 4).

**TABLE 4:** Characteristics of included articles assessing therapeutic options to prevent ferroptosis in AD stratified by mechanisms involved in ferroptosis.

Author (year)	AD model			Compound	Administration			Positive effect			
	Species	Sex	Age (year)		Form	Time (months)	Amount	Aβ	pTau	Inflamation	Cognition
Iron homeostasis											
Adlard et al., 2011	Tg2576 mice	♀	1.2	PBT2	o	0.4	30 mg/kg/d	NR	NR	NR	Y
Adlard et al., 2008	Tg2576 and APP/PS1 mice	♂, ♀	1.5–1.8	PBT2	o	0.4	30 mg/kg/d	Y	Y	NR	Y
Cherny et al., 2001	Tg2576 mice	♂, ♀	1.75	PBT1	o	2	2 mg/kg/d	Y	NR	NR	Y
Crouch et al., 2011	Aβ-induced SH−SY5Y cells	NA	NA	PBT2	NA	1 h	10–20 μM	Y	NR	NR	NA
Fine et al., 2012	TgP301L mice	NR	0.7	DFO	in	5	3 × 2.4 mg/w	Y	NR	Y	Y
Grossi et al., 2009	TgCRND8 mice	♂, ♀	0.3	PBT1	o	1.2	30 mg/kg/d	Y	NR	Y	Y
Guo et al., 2015	APP/PS1 mice	♂	0.5	DFO	in	3	200 mg/kg/2d	Y	NR	NR	NR
Guo et al., 2013	APP/PS1 mice	♂	0.5	DFO	in	3	200 mg/kg/2d	NR	Y	NR	NR
McLachlan et al., 1993	AD patients	♂, ♀	80	DFO	im	24	300 mg/d/5d/w	NR	NR	NR	Y
McLachlan et al., 1991	AD patients	♂, ♀	80	DFO	im	24	300 mg/d/5d/w	NR	NR	NR	Y
Ritchie et al., 2003	AD patients	NR	NR	PBT1	o	8.3	300–750mg/d	Y	NR	NR	Y
Glutathione metabilism											
Dumont et al., 2009	Tg19959 mice	NR	0.1	CDDO-MA	o	3	800 mg/kg chow	Y	NR	Y	Y
Fragoulis et al., 2017	APP/PS1 mice	NR	0.5	Methysticin	o	6	6 mg/kg/w	N	NR	Y	Y
Kanninen et al., 2009	APP/PS1 mice	♂	0.75	LV-Nrf2	icv	NA	2-μL	Y	NR	Y	Y
Kerr et al., 2017	ArcAβ42 flies	♂, ♀	7d	LiCl	o	NA	100 mM	Y	NR	NR	NR
Kim et al., 2013	Aβ-induced ICR mice	♂	0.4	SFN	ip	4d	30mg/kg/d	N	NR	NR	Y
Lipton et al., 2016	hAPP-J20 and 3xTg mice	NR	0.3–0.5	CA	in	3	2 × 10mg/kg/w	Y	Y	Y	Y
Nassireslami et al., 2016	Aβ-induced wistar rats	♂	NR	SA	icv	NA	5–100 nM	Y	NR	Y	Y
Wang et al., 2016	APP/PS1 mice	♂	0.3	Dl-NBP	o	5	60 mg/kg/d	Y	NR	NR	Y
Oxidative stress and lipid peroxidation											
Adair et al., 2001	AD patients	NR	NR	NAC	NR	6	50 mg/kg/day	NR	NR	NR	N
Ates et al., 2020	APPswe/PS1ΔE9 mice	♂	0.75	CMS121	o	3	34 mg/kg/d	NR	NR	Y	Y
	Aβ-induced MC65 cells	NA	NA	CMS121	NA	NR	NR	Y	NR	NR	NA
Cong et al., 2019	Aβ-induced SH−SY5Y cells	NA	NA	Chal.14a-c	NA	NA	25μM	Y	NR	NR	NA
Fu et al., 2006	Aβ-induced kunming mice	♂	0.3	NAC	ip	7d	50–200 mg/kg/d	Y	NR	NR	Y
McCaddon and Davies, 2005	AD patients	♂	65	NAC	o	NR	600 mg/d	NR	NR	NR	Y
Remington et al., 2009	AD patients	NR	NR	NAC	o	6–9	600 mg/d	NR	NR	NR	Y
Zhang et al., 2018	P301S mice	♀	0.4	LA	ip	2.3	3–10 mg/kg/d/5d/w	NR	Y	Y	Y
Zhang et al., 2017	3xTg mice	♂, ♀	0.7	Se-Met	o	3	6 μg/ml	NR	Y	NR	Y
Sripetchwandee et al., 2016	Wistar rats on HI diet	♂	0.2	DFO	ip	2	75-mg/kg/d	Y	Y	NR	NR
				NAC		2	100 mg/kg/d				

*Articles are sorted in alphabetical order and from more to less recent.*

(hAPP)-J20; mouse expressing the human amyloid precursor protein, 3xTg AD; mutant mouse with PS1M146V gene, APP/PS1; [B6C3-Tg(APPswe,PSEN1 dE9)85Dbo/J], APPswe/PS1ΔE9; transgenic mice express a mouse/human chimeric APPswe and a mutant human presinilin 1 (PS1ΔE9), ArcAβ42; Aβ42-expressing drosophila, CA; carnosic acid, Chal. 14a-c; Chalcones 14a, DFO, deferoxamine, FASN; fatty acid synthase, HI; high iron, LA; α-Lipoic acid, LV-Nrf2; human Nrf2 lentiviral vector, LiCl; lithium, N; no, NA; not applicable, NR; not reported, P301S; [B6C3-Tg (Prnp-MAPT*P301S) PS19 Vle/J], PBT1; clioquinol, SA; sodium arsenite, SFN; sulforaphane, SH-SY5Y; human neuroblastoma cells, Se-Met; selenomethionine, Tg2576; mouse line encoding human APP695 with Lys670-Asn and Met671-Leu mutations, Y; yes, d; day, icv; intracerebroventricular, im; intramuscular, in; intranasal, ip; intraperitoneal, o; oral, w; week, y; year.

### Iron Homeostasis

Our transcriptomic analysis revealed that *FTH1*, component responsible for iron storage, is differentially expressed in early and late stages of AD. Furthermore, excessive iron deposition in specific brain areas contributes to AD pathology (Antharam et al., 2012; Moon et al., 2016). Therefore, an increased interest in the development of therapeutic strategies targeting iron has emerged in the past years. In animal models, DFO treatment decreased AD hallmarks, iron overload, iron-induced kinase activity [cyclin-dependent kinase 5 (CDK5), glycogen synthase kinase 3β (GSK3β)], mitochondrial dysfunction, synaptic loss, and neuronal damage (Fine et al., 2012; Guo et al., 2013, 2015; Sripetchwandee et al., 2016). DFO increased expression of transferrin receptor (TfR1) and brain-derived neurotrophic factor (BDNF), leading to reduced iron-induced memory deficits in rodents (Fine et al., 2012; C. Guo et al., 2013, 2015; Sripetchwandee et al., 2016). In a clinical trial, DFO slowed down the progression of AD in patients (McLachlan et al., 1991, 1993). However, the dosing regimens need to be standardized before DFO could be implemented in the clinical setting (Farr and Xiong, 2021). In addition, to reduce DFO-related cytotoxicity and prolong its presence into circulation, new DFO component-containing nanogels were proposed as promising alternatives for iron-chelation in AD (Wang et al., 2018). Besides AD, DFO alone or co/treatment with ferrostatin (Fer-1, inhibitor of lipid peroxidation) also improved α-synuclein-induced pathology in a PD animal model (Febbraro et al., 2013). PBT1, a drug inhibiting zinc and copper ions from binding to Aβ, reduced Aβ deposition, attenuated astrogliosis and prevented memory impairment in AD animal models AD (Cherny et al., 2001; Grossi et al., 2009). In pilot-phase 2 clinical trial, PBT1 reduced Aβ plasma levels and, when looked specifically on severely affected AD patients, PBT1 was able to slow down the clinical decline (Ritchie et al., 2003). PBT2, a second-generation 8-hydroxyquinoline analog produced as a successor to clioquinol, induced GSK3β phosphorylation and prevented formation of Aβ in neuroblastoma SH-SY5Y cells (Crouch et al., 2011). In animal models of AD, PBT2 induced Aβ plaque degradation, decreased p-tau, rescued decreased spine density, increased brain-levels of BDNF and improved cognitive performance (Adlard et al., 2008, 2011). PBT2 was also assessed in a phase 2 clinical trial, where it lead to reduced levels of Aβ in cerebrospinal fluid and improved executive function compared to placebo (Lannfelt et al., 2008). However, PBT2 did not show any significant effect on cognition. Currently, deferiprone (DFP), a compound that alleviates symptoms related to PD pathology (Devos et al., 2014; Grolez et al., 2015; Gutbier et al., 2020), is evaluated a in phase 2 randomized placebo-controlled clinical trial with AD patients (NCT03234686). As previously reported, iron chelators can attenuate symptoms and slow down the progression of AD, which shows the potential for novel therapeutic approaches (Nuñez and Chana-Cuevas, 2018).

### Glutathione Metabolism

The revealed differential gene expression of *GPX4* and *GSS* suggests that modifying the expression or/and the activity of these gene-encoded proteins might be beneficial to treat AD. The expression of GPx4 can be directly upregulated by α-Lipoic acid (LA) (Zhang et al., 2018). LA treatment on P301S Tau transgenic mice enhanced the activity of system x_c_^–^, GPx4, superoxide dismutase 1 (Sod1), CDK5, GSK3β, TfR1 and FPN1 (Zhang et al., 2018). LA reduced the hippocampal levels of glial fibrillary acidic protein (GFAP), tumor necrosis factor α (TNF-α), interleukin 1β (IL-1β), as well as the calcium (Ca^2+^) content, p-tau, calpain1 levels, and synaptic loss. As a result, these processes led to enhanced memory function (Zhang et al., 2018). Apart from LA, GPx4 can be activated in an indirect manner through Nrf2. Nrf2 plays an important role in neurodegeneration and ferroptosis by regulating a wide range of genes (Song and Long, 2020). In addition to the activation of GPx4 and GSH synthesis (Dodson et al., 2019), it can also affect the activity of glucose-6-phosphate dehydrogenase, GSH reductase, glutamate-cysteine ligase modifier subunit (GLCM), solute carrier family 7 member 11 (SLC7A11) and others as previously summarized by Song and Long (2020). Nrf2 can be upregulated using a human lentiviral vector or compounds such as sodium arsenite, triterpenoid, 2-cyano-3,12-dioxooleana-1,9-dien-28-oic acid-methylamide (CDDO-MA), dl-3-n-butylphthalide (DI-NBP), kavalactone methysticin, carnosic acid (CA) and sulforaphane (SFN). Nrf2 upregulation increased heme oxygenase-1 (HMOX1) levels and decreased AD hallmarks, hippocampal inflammation, oxidative stress, and Aβ-induced memory deficits in AD mouse models (Dumont et al., 2009; Kanninen et al., 2009; Kim et al., 2013; Lipton et al., 2016; Nassireslami et al., 2016; Wang et al., 2016; Fragoulis et al., 2017). Finally, genetic downregulation of Kelch-like ECH-associated protein 1 (*Keap1*), the negative regulator of Nrf2, in ArcAβ42 flies, activated Nrf2, induced Aβ42 degradation, prevented neuronal toxicity in response to Aβ42 peptide, rescued neuronal-specific motor defects and increased life span (Kerr et al., 2017).

Altogether, these results suggest that inhibition of ferroptosis by targeting GSH metabolism is an important avenue for the development of new therapies for AD (Ashraf et al., 2020).

### Oxidative Stress and Lipid Peroxidation

Lipid peroxidation represents an important hallmark of AD (Sultana et al., 2013), which was also supported by the observed differential expression of *ACSL3* and 4 in the course of the pathology (Tables 1, 2). In many studies, oxidative stress was targeted to reduce neuronal damage and alleviate symptoms related to AD pathology. Anti-ferroptotic compounds that reduce oxidative stress include liproxstatin 1 (Lip-1) (inhibitor of ROS and lipid peroxidation), chalcones 14a-c (inhibitor of Aβ and lipid peroxidation), Selenomethionine (Se-Met) (inhibitor of lipid peroxidation), CMC121 (fatty acid synthase inhibitor), *N*-acetylcysteine (NAC) (free radical scavenger), Vitamin E (Vit E) and PD146176 (15-LOX-1 inhibitor). Studies using *in vitro* and *in vivo* models of AD have shown that targeting oxidative stress has a positive effect on neural degeneration, inflammation, Aβ1-42 aggregation, p-tau formation, GSH levels, iron overload, mitochondrial function, motor dysfunction and learning and memory (Fu et al., 2006; Sripetchwandee et al., 2016; Hambright et al., 2017; Zhang et al., 2017; Cong et al., 2019; Ates et al., 2020). In concordance with these results, clinical trials have shown that NAC and co-treatment of NAC, Vit E and Se-Met improved behavioral symptoms, general well-being, and neuropsychiatric and cognitive scores of AD patients (Adair et al., 2001; McCaddon and Davies, 2005; Remington et al., 2009). Although Vitamin E treatment had no beneficial effect on patients with mild cognitive impairment (Marder, 2005), it was able to improve symptoms related to other neurodegenerative diseases such as PD (Taghizadeh et al., 2017) and cerebellar ataxia (Gabsi et al., 2001). Considering the lack of adverse events of these antioxidants, ferroptosis inhibition by targeting oxidative stress is a new promising therapeutic strategy for AD.

## Discussion

Improved understanding of underlying mechanisms of ferroptosis in AD may lead to the development and application of anti-ferroptotic strategies to slow down or prevent AD progression (Han et al., 2020). Iron accumulation (Bulk et al., 2018a), lipid peroxidation (Majerníková et al., 2020) and mitochondrial dysfunction (Horowitz and Greenamyre, 2010), the main hallmarks of ferroptosis, are observed early in AD pathology, suggesting that targeting ferroptosis in AD may lead to the prevention of symptoms manifestation such as cognitive decline at advanced stages of AD.

Our analysis of DEGs in AD revealed that differential expression of ferroptosis-related genes in AD affects mostly neurons and that the changes observed in glia cells could be related to both tau phosphorylation and Aβ accumulation. This may explain the difference in the expression of ferroptotic markers between early (Aβ) and late (Aβ + p-tau) stages of AD. Even though this review has shed more light on the role of different brain cell types in ferroptosis during AD, whether ferroptosis in glia cells is related to later stages of the pathology should be investigated further.

While it is known that AD brain shows ferroptosis characteristics, it is unknown what is the causal relationship between AD and ferroptosis. Plasma ferritin increases with increasing age and Aβ deposition. Recent work on the inhibition of lipid peroxidation and iron accumulation in *C. elegans* revealed extended life- and health-span independently of other mechanisms (Jenkins et al., 2020). This evidence suggests that ferroptosis may be an age-related as well as disease-related process (Goozee et al., 2018; Larric et al., 2020). Therefore, ferroptosis inhibition may not only lead to slowing down the neurodegeneration but also contribute to longer health-span (Larric et al., 2020).

Iron dysregulation aggravates formation and aggregation of both Aβ and p-tau protein forming plaques and NFT respectively (Derry et al., 2020). Even though the link between ferroptosis and Aβ has been extensively studied, much less is known about its role in NFT formation. Therefore, future studies should try to investigate the role of ferroptosis in hyperphosphorylation of tau protein and formation of fibrillary tangles independently of Aβ pathology. This could be achieved by comparing the characteristics of ferroptotic cell death in AD with patients with primary age-related tauopathy (PART) (Crary et al., 2014).

Further research should also address the effect of ferroptosis on the interactions between different cell types in AD context. Although cell-cell interactions are dysregulated in AD brain (Henstridge et al., 2019), this feature of AD is often overlooked in *in vitro* studies. The brain-on-a-chip platform using induced pluripotent stem cells (iPSCs) -derived neurons and glia from AD patients could allow a high throughput screening of the effect of anti-ferroptotic drugs in AD, while mimicking the cell-cell interactions in AD context (Trombetta-Lima et al., 2021). Moreover, this model is easily reproducible and thanks to the use of iPSCs from AD patients, also more translatable to humans compared to well-established animal models.

## Conclusion

This review summarizes the evidence supporting the important role of ferroptosis in AD pathology and presents what is known about the targets for its inhibition for a potential treatment. Ferroptosis-related genes are differentially expressed in AD, supporting our hypothesis that ferroptosis inhibition could slow down the AD progression and memory decline, however, many questions remain unanswered. Developing new AD models allowing us to study how ferroptosis effects cell-cell interaction is needed to understand the causal relationship and timing of ferroptosis in AD. Future efforts should be directed toward developing detection techniques of ferroptosis *in vivo* and organizing large, randomized clinical trials of anti-ferroptotic drugs in early and late stages of AD progression.

## Author Contributions

NM, AD, and WD designed the theme of the manuscript. NM contributed by writing all the sections and creating all tables and figures. AD and WD conducted critical revisions of the manuscript. All authors contributed to the article and approved the submitted version.

## Conflict of Interest

The authors declare that the research was conducted in the absence of any commercial or financial relationships that could be construed as a potential conflict of interest.

## Publisher’s Note

All claims expressed in this article are solely those of the authors and do not necessarily represent those of their affiliated organizations, or those of the publisher, the editors and the reviewers. Any product that may be evaluated in this article, or claim that may be made by its manufacturer, is not guaranteed or endorsed by the publisher.

## References

[B1] Acosta-Cabronero J. Betts M. J. Cardenas-Blanco A. Yang S. Nestor P. J. (2016). In vivo MRI mapping of brain iron deposition across the adult lifespan. *J. Neurosci.* 36 364–374. 10.1523/JNEUROSCI.1907-15.2016 26758829 PMC4710766

[B2] Adair J. C. Knoefel J. E. Morgan N. (2001). Controlled trial of N-acetylcysteine for patients with probable Alzheimer’s disease. *Neurology* 57 1515–1517. 10.1212/WNL.57.8.1515 11673605

[B3] Adlard P. A. Bica L. White A. R. Nurjono M. Filiz G. Crouch P. J. (2011). Metal ionophore treatment restores dendritic spine density and synaptic protein levels in a mouse model of Alzheimer’s disease. *PLoS One* 6:e17669. 10.1371/journal.pone.0017669 21412423 PMC3055881

[B4] Adlard P. A. Cherny R. A. Finkelstein D. I. Gautier E. Robb E. Cortes M. (2008). Rapid Restoration of Cognition in Alzheimer’s Transgenic Mice with 8-Hydroxy Quinoline Analogs Is Associated with Decreased Interstitial Aβ. *Neuron* 59 43–55. 10.1016/j.neuron.2008.06.018 18614028

[B5] Altamura S. Muckenthaler M. U. (2009). Iron toxicity in diseases of aging: Alzheimer’s disease, Parkinson’s disease and atherosclerosis. *J. Alzheimer’s Dis.* 16 879–895. 10.3233/JAD-2009-1010 19387120

[B6] Antharam V. Collingwood J. F. Bullivant J. P. Davidson M. R. Chandra S. Mikhaylova A. (2012). High field magnetic resonance microscopy of the human hippocampus in Alzheimer’s disease: Quantitative imaging and correlation with iron. *NeuroImage* 59 1249–1260. 10.1016/j.neuroimage.2011.08.019 21867761 PMC3690369

[B7] Apostolakis S. Kypraiou A. M. (2017). Iron in neurodegenerative disorders: Being in the wrong place at the wrong time? *Rev. Neurosci.* 28 893–911. 10.1515/revneuro-2017-0020 28792913

[B8] Aprioku J. S. (2013). Pharmacology of free radicals and the impact of reactive oxygen species on the testis. *J. Reproduct. Infertil.* 14 158–172. PMC3911811 24551570

[B9] Ashraf A. Jeandriens J. Parkes H. G. So P. W. (2020). Iron dyshomeostasis, lipid peroxidation and perturbed expression of cystine/glutamate antiporter in Alzheimer’s disease: Evidence of ferroptosis. *Redox Biol.* 32:101494. 10.1016/j.redox.2020.101494 32199332 PMC7083890

[B10] Ates G. Goldberg J. Currais A. Maher P. (2020). CMS121, a fatty acid synthase inhibitor, protects against excess lipid peroxidation and inflammation and alleviates cognitive loss in a transgenic mouse model of Alzheimer’s disease. *Redox Biol.* 36:101648. 10.1016/j.redox.2020.101648 32863221 PMC7394765

[B11] Ayton S. Fazlollahi A. Bourgeat P. Raniga P. Ng A. Lim Y. Y. (2017). Cerebral quantitative susceptibility mapping predicts amyloid-β-related cognitive decline. *Brain* 140 2112–2119. 10.1093/brain/awx137 28899019

[B12] Barnham K. J. McKinstry W. J. Multhaup G. Galatis D. Morton C. J. Curtain C. C. (2003). Structure of the Alzheimer’s disease amyloid precursor protein copper binding domain. A regulator of neuronal copper homeostasis. *J. Biol. Chem.* 278 17401–17407. 10.1074/jbc.M300629200 12611883

[B13] Becerril-Ortega J. Bordji K. Fréret T. Rush T. Buisson A. (2014). Iron overload accelerates neuronal amyloid-β production and cognitive impairment in transgenic mice model of Alzheimer’s disease. *Neurobiol. Aging* 35 2288–2301. 10.1016/j.neurobiolaging.2014.04.019 24863668

[B14] Birben E. Sahiner U. M. Sackesen C. Erzurum S. Kalayci O. (2012). Oxidative stress and antioxidant defense. *World Allergy Organizat. J.* 5 9–19. 10.1097/WOX.0b013e3182439613 23268465 PMC3488923

[B15] Bradley-Whitman M. A. Lovell M. A. (2015). Biomarkers of lipid peroxidation in Alzheimer disease (AD): an update. *Arch. Toxicol.* 89 1035–1044. 10.1007/s00204-015-1517-6 25895140 PMC4466146

[B16] Bulk M. Kenkhuis B. Van Der Graaf L. M. Goeman J. J. Natté R. Van Der Weerd L. (2018b). Postmortem T2*-Weighted MRI Imaging of Cortical Iron Reflects Severity of Alzheimer’s Disease. *J. Alzheimer’s Dis.* 65 1125–1137. 10.3233/JAD-180317 30103327 PMC6218127

[B17] Bulk M. Abdelmoula W. M. Nabuurs R. J. A. van der Graaf L. M. Mulders C. W. H. Mulder A. A. (2018a). Postmortem MRI and histology demonstrate differential iron accumulation and cortical myelin organization in early- and late-onset Alzheimer’s disease. *Neurobiol. Aging* 62 231–242. 10.1016/j.neurobiolaging.2017.10.017 29195086

[B18] Bush A. I. (2013). The metal theory of Alzheimer’s disease. *J. Alzheimer’s Dis.* 33(Suppl. 1), S277–S281. 10.3233/JAD-2012-129011 22635102

[B19] Castellani R. J. Moreira P. I. Liu G. Dobson J. Perry G. Smith M. A. (2007). Iron: The redox-active center of oxidative stress in Alzheimer disease. *Neurochem. Res.* 32 1640–1645. 10.1007/s11064-007-9360-7 17508283

[B20] Chang Y. (2019). Cellulat iron metabolism and regulation. *Brain Iron Metabol. CNS Dis.* 1173 21–32. 10.1007/978-981-13-9589-5_2 31456203

[B21] Chen W. T. Lu A. Craessaerts K. Pavie B. Sala Frigerio C. Corthout N. (2020). Spatial Transcriptomics and In Situ Sequencing to Study Alzheimer’s Disease. *Cell* 182 976.e–991.e. 10.1016/j.cell.2020.06.038 32702314

[B22] Cherny R. A. Atwood C. S. Xilinas M. E. Gray D. N. Jones W. D. McLean C. A. (2001). Treatment with a copper-zinc chelator markedly and rapidly inhibits β-amyloid accumulation in Alzheimer’s disease transgenic mice. *Neuron* 30 665–676. 10.1016/S0896-6273(01)00317-8 11430801

[B23] Cong L. Dong X. Wang Y. Deng Y. Li B. Dai R. (2019). On the role of synthesized hydroxylated chalcones as dual functional amyloid-β aggregation and ferroptosis inhibitors for potential treatment of Alzheimer’s disease. *Eur. J. Med. Chem.* 166 11–21. 10.1016/j.ejmech.2019.01.039 30684867

[B24] Cozza G. Rossetto M. Bosello-Travain V. Maiorino M. Roveri A. Toppo S. (2017). Glutathione peroxidase 4-catalyzed reduction of lipid hydroperoxides in membranes: The polar head of membrane phospholipids binds the enzyme and addresses the fatty acid hydroperoxide group toward the redox center. *Free Radic. Biol. Med.* 112 1–11. 10.1016/j.freeradbiomed.2017.07.010 28709976

[B25] Crary J. F. Trojanowski J. Q. Schneider J. A. Abisambra J. F. Abner E. L. Alafuzoff I. (2014). Primary age-related tauopathy (PART): a common pathology associated with human aging. *Acta Neuropathol.* 128 755–766. 10.1007/s00401-014-1349-0 25348064 PMC4257842

[B26] Crouch P. J. Savva M. S. Hung L. W. Donnelly P. S. Mot A. I. Parker S. J. (2011). The Alzheimer’s therapeutic PBT2 promotes amyloid-β degradation and GSK3 phosphorylation via a metal chaperone activity. *J. Neurochem.* 119 220–230. 10.1111/j.1471-4159.2011.07402.x 21797865

[B27] da Rocha T. J. Silva Alves M. Guisso C. C. de Andrade F. M. Camozzato A. de Oliveira A. A. (2018). Association of GPX1 and GPX4 polymorphisms with episodic memory and Alzheimer’s disease. *Neurosci. Lett.* 666 32–37. 10.1016/j.neulet.2017.12.026 29246792

[B28] DeHart D. N. Fang D. Heslop K. Li L. Lemasters J. J. Maldonado E. N. (2018). Opening of voltage dependent anion channels promotes reactive oxygen species generation, mitochondrial dysfunction and cell death in cancer cells. *Biochem. Pharmacol.* 148 155–162. 10.1016/j.bcp.2017.12.022 29289511 PMC5909406

[B29] Deibel M. A. Ehmann W. D. Markesbery W. R. (1996). Copper, iron, and zinc imbalances in severely degenerated brain regions in Alzheimer’s disease: Possible relation to oxidative stress. *J. Neurol. Sci.* 143 137–142. 10.1016/S0022-510X(96)00203-1 8981312

[B30] Derry P. J. Hegde M. L. Jackson G. R. Kayed R. Tour J. M. Tsai A. L. (2020). Revisiting the intersection of amyloid, pathologically modified tau and iron in Alzheimer’s disease from a ferroptosis perspective. *Prog. Neurobiol.* 184:101716. 10.1016/j.pneurobio.2019.101716 31604111 PMC7850812

[B31] Devos D. Moreau C. Devedjian J. C. Kluza J. Petrault M. Laloux C. (2014). Targeting chelatable iron as a therapeutic modality in Parkinson’s disease. *Antioxid. Redox Signal.* 21 195–210. 10.1089/ars.2013.5593 24251381 PMC4060813

[B32] Dixon S. J. (2017). Ferroptosis: bug or feature? *Immunol. Rev.* 277 150–157. 10.1111/imr.12533 28462529

[B33] Dixon S. J. Lemberg K. M. Lamprecht M. R. Skouta R. Zaitsev E. M. Gleason C. E. (2012). Ferroptosis: An iron-dependent form of nonapoptotic cell death. *Cell* 149 1060–1072. 10.1016/j.cell.2012.03.042 22632970 PMC3367386

[B34] Dixon S. J. Patel D. Welsch M. Skouta R. Lee E. Hayano M. (2014). Pharmacological inhibition of cystine-glutamate exchange induces endoplasmic reticulum stress and ferroptosis. *ELife* 3:e02523. 10.7554/eLife.02523 24844246 PMC4054777

[B35] Dodson M. Castro-Portuguez R. Zhang D. D. (2019). NRF2 plays a critical role in mitigating lipid peroxidation and ferroptosis. *Redox Biol.* 23:101107. 10.1016/j.redox.2019.101107 30692038 PMC6859567

[B36] Du L. Zhao Z. Cui A. Zhu Y. Zhang L. Liu J. (2018). Increased Iron Deposition on Brain Quantitative Susceptibility Mapping Correlates with Decreased Cognitive Function in Alzheimer’s Disease. *ACS Chemical Neurosci.* 9 1849–1857. 10.1021/acschemneuro.8b00194 29722955

[B37] Dugger B. N. Dickson D. W. (2017). Pathology of neurodegenerative diseases. *Cold Spring Harb. Perspect. Biol.* 9:a028035. 10.1101/cshperspect.a028035 28062563 PMC5495060

[B38] Dumont M. Wille E. Calingasan N. Y. Tampellini D. Williams C. Gouras G. K. (2009). Triterpenoid CDDO-methylamide improves memory and decreases amyloid plaques in a transgenic mouse model of Alzheimer’s disease. *J. Neurochem.* 109 502–512. 10.1111/j.1471-4159.2009.05970.x 19200343 PMC3083825

[B39] Eleftheriadis N. Poelman H. Leus N. G. J. Honrath B. Neochoritis C. G. Dolga A. (2016). Design of a novel thiophene inhibitor of 15-lipoxygenase-1 with both anti-inflammatory and neuroprotective properties. *Eur. J. Med. Chem.* 122, 786–801. 10.1016/j.ejmech.2016.07.010 27477687 PMC5010146

[B40] Farr A. C. Xiong M. P. (2021). Challenges and Opportunities of Deferoxamine Delivery for Treatment of Alzheimer’s Disease, Parkinson’s Disease, and Intracerebral Hemorrhage. *Mol. Pharmaceut.* 18 593–609. 10.1021/acs.molpharmaceut.0c00474 32926630 PMC8819678

[B41] Febbraro F. Andersen K. J. Sanchez-Guajardo V. Tentillier N. Romero-Ramos M. (2013). Chronic intranasal deferoxamine ameliorates motor defects and pathology in the α-synuclein rAAV Parkinson’s model. *Exp. Neurol.* 247 45–58. 10.1016/j.expneurol.2013.03.017 23531432

[B42] Fine J. M. Baillargeon A. M. Renner D. B. Hoerster N. S. Tokarev J. Colton S. (2012). Intranasal deferoxamine improves performance in radial arm water maze, stabilizes HIF-1α, and phosphorylates GSK3β in P301L tau transgenic mice. *Exp. Brain Res.* 219 381–390. 10.1007/s00221-012-3101-0 22547371

[B43] Fragoulis A. Siegl S. Fendt M. Jansen S. Soppa U. Brandenburg L. O. (2017). Oral administration of methysticin improves cognitive deficits in a mouse model of Alzheimer’s disease. *Redox Biol.* 12 843–853. 10.1016/j.redox.2017.04.024 28448946 PMC5406548

[B44] Fu A. L. Dong Z. H. Sun M. J. (2006). Protective effect of N-acetyl-l-cysteine on amyloid β-peptide-induced learning and memory deficits in mice. *Brain Res.* 1109 201–206. 10.1016/j.brainres.2006.06.042 16872586

[B45] Gabsi S. Gouider-Khouja N. Belal S. Fki M. Kefi M. Turki I. (2001). Effect of vitamin E supplementation in patients with ataxia with vitamin E deficiency. *Eur. J. Neurol.* 8 477–481. 10.1046/j.1468-1331.2001.00273.x 11554913

[B46] Gao M. Yi J. Zhu J. Minikes A. M. Monian P. Thompson C. B. (2019). Role of Mitochondria in Ferroptosis. *Mol. Cell* 73 354.e–363.e. 10.1016/j.molcel.2018.10.042 30581146 PMC6338496

[B47] Gaugler J. James B. Johnson T. Scholz K. Weuve J. (2016). 2016 Alzheimer’s disease facts and figures. *Alzheimer’s Dement.* 2 459–509. 10.1016/j.jalz.2016.03.001 27570871

[B48] Gerrits E. Brouwer N. Kooistra S. M. Woodbury M. E. Vermeiren Y. Lambourne M. (2021). Distinct amyloid-β and tau-associated microglia profiles in Alzheimer’s disease. *Acta Neuropathol.* 141 681–696. 10.1007/s00401-021-02263-w 33609158 PMC8043951

[B49] Goozee K. Chatterjee P. James I. Shen K. Sohrabi H. R. Asih P. R. (2018). Elevated plasma ferritin in elderly individuals with high neocortical amyloid-β load. *Mol. Psychiatry* 23 1807–1812. 10.1038/mp.2017.146 28696433

[B50] Griffith O. W. (1982). Mechanism of action, metabolism, and toxicity of buthionine sulfoximine and its higher homologs, potent inhibitors of glutathione synthesis. *J. Biol. Chem.* 257 13704–13712. 6128339

[B51] Grolez G. Moreau C. Sablonnière B. Garçon G. Devedjian J. C. Meguig S. (2015). Ceruloplasmin activity and iron chelation treatment of patients with Parkinson’s disease. *BMC Neurol.* 6:74. 10.1186/s12883-015-0331-3 25943368 PMC4429376

[B52] Grossi C. Francese S. Casini A. Rosi M. C. Luccarini I. Fiorentini A. (2009). Clioquinol decreases amyloid-β burden and reduces working memory impairment in a transgenic mouse model of alzheimer’s disease. *J. Alzheimer’s Dis.* 17 423–440. 10.3233/JAD-2009-1063 19363260

[B53] Guo C. Wang P. Zhong M. L. Wang T. Huang X. S. Li J. Y. (2013). Deferoxamine inhibits iron induced hippocampal tau phosphorylation in the Alzheimer transgenic mouse brain. *Neurochem. Int.* 62 165–172. 10.1016/j.neuint.2012.12.005 23262393

[B54] Guo C. Zhang Y. X. Wang T. Zhong M. L. Yang Z. H. Hao L. J. (2015). Intranasal deferoxamine attenuates synapse loss via up-regulating the P38/HIF-1α pathway on the brain of APP/PS1 transgenic mice. *Front. Aging Neurosci.* 7:104. 10.3389/fnagi.2015.00104 26082716 PMC4451419

[B55] Guo X. Lin H. Liu J. Yao P. (2019). Quercetin Protects Hepatocyte from Ferroptosis by Depressing Mitochondria-reticulum Interaction Through PERK Downregulation in Alcoholic Liver (P06-056-19). *Curr. Dev. Nutrit.* 2019:19. 10.1093/cdn/nzz031.p06-056-19

[B56] Gutbier S. Kyriakou S. Schildknecht S. Ückert A. K. Brüll M. Lewis F. (2020). Design and evaluation of bi-functional iron chelators for protection of dopaminergic neurons from toxicants. *Arch. Toxicol.* 94 3105–3123. 10.1007/s00204-020-02826-y 32607613 PMC7415766

[B57] Habib E. Linher-Melville K. Lin H. X. Singh G. (2015). Expression of xCT and activity of system xc- are regulated by NRF2 in human breast cancer cells in response to oxidative stress. *Redox Biol.* 5 33–42. 10.1016/j.redox.2015.03.003 25827424 PMC4392061

[B58] Hambright W. S. Fonseca R. S. Chen L. Na R. Ran Q. (2017). Ablation of ferroptosis regulator glutathione peroxidase 4 in forebrain neurons promotes cognitive impairment and neurodegeneration. *Redox Biol.* 12 8–17. 10.1016/j.redox.2017.01.021 28212525 PMC5312549

[B59] Han C. Liu Y. Dai R. Ismail N. Su W. Li B. (2020). Ferroptosis and Its Potential Role in Human Diseases. *Front. Pharmacol.* 11:239. 10.3389/fphar.2020.00239 32256352 PMC7090218

[B60] Heneka M. T. McManus R. M. Latz E. (2018). Inflammasome signalling in brain function and neurodegenerative disease. *Nat. Rev. Neurosci.* 19 610–621. 10.1038/s41583-018-0055-7 30206330

[B61] Henstridge C. M. Hyman B. T. Spires-Jones T. L. (2019). Beyond the neuron–cellular interactions early in Alzheimer disease pathogenesis. *Nat. Rev. Neurosci.* 20 94–108. 10.1038/s41583-018-0113-1 30643230 PMC6545070

[B62] Horowitz M. P. Greenamyre J. T. (2010). Mitochondrial iron metabolism and its role in neurodegeneration. *J. Alzheimer’s Dis.* 20(Suppl. 2), S551–S568. 10.3233/JAD-2010-100354 20463401 PMC3085540

[B63] Hou W. Xie Y. Song X. Sun X. Lotze M. T. Zeh H. J. (2016). Autophagy promotes ferroptosis by degradation of ferritin. *Autophagy* 12 1425–1428. 10.1080/15548627.2016.1187366 27245739 PMC4968231

[B64] Huang W. J. Zhang X. Chen W. W. (2016). Role of oxidative stress in Alzheimer’s disease. *Biomed. Rep.* 4 519–522. 10.3892/br.2016.630 27123241 PMC4840676

[B65] James S. A. Churches Q. I. De Jonge M. D. Birchall I. E. Streltsov V. McColl G. (2017). Iron, Copper, and Zinc Concentration in Aβ Plaques in the APP/PS1 Mouse Model of Alzheimer’s Disease Correlates with Metal Levels in the Surrounding Neuropil. *ACS Chem. Neurosci.* 8 629–637. 10.1021/acschemneuro.6b00362 27958708

[B66] Jelinek A. Heyder L. Daude M. Plessner M. Krippner S. Grosse R. (2018). Mitochondrial rescue prevents glutathione peroxidase-dependent ferroptosis. *Free Radic. Biol. Med.* 117 45–57. 10.1016/j.freeradbiomed.2018.01.019 29378335

[B67] Jenkins N. L. James S. A. Salim A. Sumardy F. Speed T. P. Conrad M. (2020). Changes in ferrous iron and glutathione promote ferroptosis and frailty in aging caenorhabditis elegans. *ELife* 9:e56580. 10.7554/eLife.56580 32690135 PMC7373428

[B68] Jiang L. Kon N. Li T. Wang S. J. Su T. Hibshoosh H. (2015). Ferroptosis as a p53-mediated activity during tumour suppression. *Nature* 520 57–62. 10.1038/nature14344 25799988 PMC4455927

[B69] Jiang T. Cheng H. Su J. Wang X. Wang Q. Chu J. (2020). Gastrodin protects against glutamate-induced ferroptosis in HT-22 cells through Nrf2/HO-1 signaling pathway. *Toxicol. Vitro* 2020:104715. 10.1016/j.tiv.2019.104715 31698019

[B70] Joo E. Yoon S. Chung H. Sharma N. Trong B. Sung N. (2020). Glutathione Peroxidase - 1 Knockout Facilitates Memory Impairment Induced by β - Amyloid (1 – 42) in Mice via Inhibition of PKC βII - Mediated ERK Signaling; Application with Glutathione Peroxidase - 1 Gene - Encoded Adenovirus Vector. *Neurochem. Res.* 2020:0123456789. 10.1007/s11064-020-03147-3 33064252

[B71] Kagan V. E. Mao G. Qu F. Angeli J. P. F. Doll S. Croix C. S. (2017). Oxidized arachidonic and adrenic PEs navigate cells to ferroptosis. *Nat. Chem. Biol.* 13 81–90. 10.1038/nchembio.2238 27842066 PMC5506843

[B72] Kanninen K. Heikkinen R. Malm T. Rolova T. Kuhmonen S. Leinonen H. (2009). Intrahippocampal injection of a lentiviral vector expressing Nrf2 improves spatial learning in a mouse model of Alzheimer’s disease. *Proc. Natl. Acad. Sci. U S A.* 106 16505–16510. 10.1073/pnas.0908397106 19805328 PMC2752553

[B73] Karch C. M. Ezerskiy L. A. Bertelsen S. Goate A. M. Albert M. S. Albin R. L. (2016). Alzheimer’s disease risk polymorphisms regulate gene expression in the ZCWPW1 and the CELF1 loci. *PLoS One* 11:e0148717. 10.1371/journal.pone.0148717 26919393 PMC4769299

[B74] Kaufmann M. R. Barth S. Konietzko U. Wu B. Egger S. Kunze R. (2013). Dysregulation of hypoxia-inducible factor by presenilin/γ-secretase loss-of-function mutations. *J. Neurosci.* 33 1915–1926. 10.1523/JNEUROSCI.3402-12.2013 23365231 PMC6619134

[B75] Kerr F. Sofola-Adesakin O. Ivanov D. K. Gatliff J. Gomez Perez-Nievas B. Bertrand H. C. (2017). Direct Keap1-Nrf2 disruption as a potential therapeutic target for Alzheimer’s disease. *PLoS Genet.* 13:e1006593. 10.1371/journal.pgen.1006593 28253260 PMC5333801

[B76] Kim H. V. Kim H. Y. Ehrlich H. Y. Choi S. Y. Kim D. J. Kim Y. S. (2013). Amelioration of Alzheimer’s disease by neuroprotective effect of sulforaphane in animal model. *Amyloid* 20 7–12. 10.3109/13506129.2012.751367 23253046

[B77] Kim S. Kim Y. Kim S. E. An J. (2021). Ferroptosis-Related Genes in Neurodevelopment and Central Nervous System. *Biology* 10:35. 10.3390/biology10010035 33419148 PMC7825574

[B78] Krabbendam I. E. Honrath B. Dilberger B. Iannetti E. F. Branicky R. S. Meyer T. (2020). SK channel-mediated metabolic escape to glycolysis inhibits ferroptosis and supports stress resistance in C. elegans. *Cell Death Dis.* 11:263. 10.1038/s41419-020-2458-4 32327637 PMC7181639

[B79] Kuang F. Liu J. Tang D. Kang R. (2020). Oxidative Damage and Antioxidant Defense in Ferroptosis. *Front. Cell Dev. Biol.* 2020:1–10. 10.3389/fcell.2020.586578 33043019 PMC7527737

[B80] Lachaier E. Louandre C. Godin C. Saidak Z. Baert M. Diouf M. (2014). Sorafenib induces ferroptosis in human cancer cell lines originating from different solid tumors. *Anticancer Res.* 34 6417–6422. 25368241

[B81] Langkammer C. Ropele S. Pirpamer L. Fazekas F. Schmidt R. (2014). MRI for iron mapping in Alzheimer’s disease. *Neurodegenerat. Dis.* 13 189–191. 10.1159/000353756 23942230

[B82] Lannfelt L. Blennow K. Zetterberg H. Batsman S. Ames D. Harrison J. (2008). Safety, efficacy, and biomarker findings of PBT2 in targeting Aβ as a modifying therapy for Alzheimer’s disease: a phase IIa, double-blind, randomised, placebo-controlled trial. *Lancet Neurol.* 7 779–786. 10.1016/S1474-4422(08)70167-4 18672400

[B83] Larric J. W. Larric J. W. Mendelsoh A. R. (2020). Contribution of Ferroptosis to Aging and Frailty. *Rejuvenat. Res.* 23 434–438. 10.1089/rej.2020.2390 32977738

[B84] Lee J. H. Lee M. S. (2019). Brain iron accumulation in atypical parkinsonian syndromes: In vivo MRI evidences for distinctive patterns. *Front. Neurol.* 10:74. 10.3389/fneur.2019.00074 30809185 PMC6379317

[B85] Li J. Cao F. Yin H. I Huang Z. J. Lin Z. T. Mao N. (2020). Ferroptosis: past, present and future. *Cell Death Dis.* 11:2. 10.1038/s41419-020-2298-2 32015325 PMC6997353

[B86] Li Q. Sun M. (2017). The role of autophagy in Alzheimer’s disease. *J. Syst. Integrat. Neurosci.* 3 1–6. 10.15761/jsin.1000172

[B87] Liang C. Zhang X. Yang M. Dong X. (2019). Recent Progress in Ferroptosis Inducers for Cancer Therapy. *Adv. Mater.* 31:e1904197. 10.1002/adma.201904197 31595562

[B88] Lipton S. A. Rezaie T. Nutter A. Lopez K. M. Parker J. Kosaka K. (2016). Therapeutic advantage of pro-electrophilic drugs to activate the Nrf2/ARE pathway in Alzheimer’s disease models. *Cell Death Dis.* 7:389. 10.1038/cddis.2016.389 27906174 PMC5261011

[B89] Lovell M. A. Xie C. Markesbery W. R. (2001). Acrolein is increased in Alzheimer’s disease brain and is toxic to primary hippocampal cultures. *Neurobiol. Aging* 22 187–194. 10.1016/S0197-4580(00)00235-9 11182468

[B90] Maher P. van Leyen K. Dey P. N. Honrath B. Dolga A. Methner A. (2018). The role of Ca2+ in cell death caused by oxidative glutamate toxicity and ferroptosis. *Cell Calcium* 70 47–55. 10.1016/j.ceca.2017.05.007 28545724 PMC5682235

[B91] Majerníková N. Jia J. Andrea Y. (2020). CuATSM PET to diagnose age - related diseases: a systematic literature review. *Clin. Translat. Imaging* 8 449–460. 10.1007/s40336-020-00394-w

[B92] Marder K. (2005). Vitamin E and donepezil for the treatment of mild cognitive impairment. *Curr. Neurol. Neurosci. Rep.* 5 337–338. 10.1007/s11910-005-0056-6 16131415

[B93] Marmolejo-Garza A. Dolga A. M. (2021). PEG out through the pores with the help of ESCRTIII. *Cell Calcium* 97:102422. 10.1016/j.ceca.2021.102422 34098170

[B94] Masaldan S. Belaidi A. A. Ayton S. Bush A. I. (2019). Cellular senescence and iron dyshomeostasis in alzheimer’s disease. *Pharmaceuticals* 12:93. 10.3390/ph12020093 31248150 PMC6630536

[B95] Mathys H. Davila-Velderrain J. Peng Z. Gao F. Mohammadi S. Young J. Z. (2019). Single-cell transcriptomic analysis of Alzheimer’s disease. *Nature* 570 332–337. 10.1038/s41586-019-1195-2 31042697 PMC6865822

[B96] McCaddon A. Davies G. (2005). Co-administration of N-acetylcysteine, vitamin B12 and folate in cognitively impaired hyperhomocysteinaemic patients. *Int. J. Geriatr. Psychiatry* 20 998–1000. 10.1002/gps.1376 16173005

[B97] McLachlan D. R. C. Kruck T. P. A. Kalow W. Andrews D. F. Dalton A. J. Bell M. Y. (1991). Intramuscular desferrioxamine in patients with Alzheimer’s disease. *Lancet* 337 1304–1308. 10.1016/0140-6736(91)92978-B 1674295

[B98] McLachlan D. R. Smith W. L. Kruck T. P. (1993). Desferrioxamine and alzheimer’s disease: Video home behavior assessment of clinical course and measures of brain aluminum. *Therapeut. Drug Monitor.* 15 602–607. 10.1097/00007691-199312000-00027 8122302

[B99] Montine T. J. Markesbery W. R. Morrow J. D. Roberts L. J. (1998). Cerebrospinal fluid F2-isoprostane levels are increased in Alzheimer’s disease. *Ann. Neurol.* 44 410–413. 10.1002/ana.410440322 9749613

[B100] Moon Y. Han S. H. Moon W. J. (2016). Patterns of Brain Iron Accumulation in Vascular Dementia and Alzheimer’s Dementia Using Quantitative Susceptibility Mapping Imaging. *J. Alzheimer’s Dis.* 51 737–745. 10.3233/JAD-151037 26890777

[B101] Nassireslami E. Nikbin P. Amini E. Payandemehr B. Shaerzadeh F. Khodagholi F. (2016). How sodium arsenite improve amyloid β-induced memory deficit? *Physiol. Behav.* 163 97–106. 10.1016/j.physbeh.2016.04.046 27129674

[B102] Neitemeier S. Jelinek A. Laino V. Hoffmann L. Eisenbach I. Eying R. (2017). BID links ferroptosis to mitochondrial cell death pathways. *Redox Biol.* 12 558–570. 10.1016/j.redox.2017.03.007 28384611 PMC5382034

[B103] Nuñez M. T. Chana-Cuevas P. (2018). New perspectives in iron chelation therapy for the treatment of neurodegenerative diseases. *Pharmaceuticals* 11:109. 10.3390/ph11040109 30347635 PMC6316457

[B104] Obulesu M. Lakshmi M. J. (2014). Apoptosis in Alzheimer’s Disease: An Understanding of the Physiology, Pathology and Therapeutic Avenues. *Neurochem. Res.* 39 2301–2312. 10.1007/s11064-014-1454-4 25322820

[B105] Peters D. G. Pollack A. N. Cheng K. C. Sun D. Saido T. Haaf M. P. (2018). Dietary lipophilic iron alters amyloidogenesis and microglial morphology in Alzheimer’s disease knock-in APP mice. *Metallomics* 10 426–443. 10.1039/c8mt00004b 29424844

[B106] Picca A. Mankowski R. T. Kamenov G. Anton S. D. Manini T. M. Buford T. W. (2019). Advanced Age Is Associated with Iron Dyshomeostasis and Mitochondrial DNA Damage in Human Skeletal Muscle. *Cells* 8:1525. 10.3390/cells8121525 31783583 PMC6953082

[B107] Plascencia-Villa G. Ponce A. Collingwood J. F. Josefina Arellano-Jiménez M. Zhu X. Rogers J. T. (2016). High-resolution analytical imaging and electron holography of magnetite particles in amyloid cores of Alzheimer’s disease. *Sci. Rep.* 6 1–12. 10.1038/srep24873 27121137 PMC4848473

[B108] Praticò D. Sung S. (2004). Lipid Peroxidation and Oxidative imbalance: Early functional events in Alzheimer’s disease. *J. Alzheimer’s Dis.* 6 171–175. 10.3233/JAD-2004-6209 15096701

[B109] Praticò D. Uryu K. Leight S. Trojanoswki J. Q. Lee V. M. Y. (2001). Increased lipid peroxidation precedes amyloid plaque formation in an animal model of alzheimer amyloidosis. *J. Neurosci.* 21 4183–4187. 10.1523/jneurosci.21-12-04183.2001 11404403 PMC6762743

[B110] Remington R. Chan A. Paskavitz J. Shea T. B. (2009). Efficacy of a vitamin/nutriceutical formulation for moderate-stage to later-stage alzheimer’s disease: A placebo-controlled pilot study. *Am. J. Alzheimer’s Dis. Dement.* 24 27–33. 10.1177/1533317508325094 19056706 PMC10846219

[B111] Ritchie C. W. Bush A. I. Mackinnon A. Macfarlane S. Mastwyk M. MacGregor L. (2003). Metal-Protein Attenuation with Iodochlorhydroxyquin (Clioquinol) Targeting Aβ Amyloid Deposition and Toxicity in Alzheimer Disease: A Pilot Phase 2 Clinical Trial. *Arch. Neurol.* 60 1685–1691. 10.1001/archneur.60.12.1685 14676042

[B112] Sato H. Nomura S. Maebara K. Sato K. Tamba M. Bannai S. (2004). Transcriptional control of cystine/glutamate transporter gene by amino acid deprivation. *Biochem. Biophys. Res. Commun.* 325 109–116. 10.1016/j.bbrc.2004.10.009 15522208

[B113] Sato M. Kusumi R. Hamashima S. Kobayashi S. Sasaki S. Komiyama Y. (2018). The ferroptosis inducer erastin irreversibly inhibits system xc- and synergizes with cisplatin to increase cisplatin’s cytotoxicity in cancer cells. *Sci. Rep.* 8:968. 10.1038/s41598-018-19213-4 29343855 PMC5772355

[B114] Seibt T. M. Proneth B. Conrad M. (2019). Role of GPX4 in ferroptosis and its pharmacological implication. *Free Radic. Biol. Med.* 133 144–152. 10.1016/j.freeradbiomed.2018.09.014 30219704

[B115] Seiler A. Schneider M. Förster H. Roth S. Wirth E. K. Culmsee C. (2008). Glutathione Peroxidase 4 Senses and Translates Oxidative Stress into 12/15-Lipoxygenase Dependent- and AIF-Mediated Cell Death. *Cell Metabol.* 8 237–248. 10.1016/j.cmet.2008.07.005 18762024

[B116] Shah R. Shchepinov M. S. Pratt D. A. (2018). Resolving the Role of Lipoxygenases in the Initiation and Execution of Ferroptosis. *ACS Central Sci.* 4 387–396. 10.1021/acscentsci.7b00589 29632885 PMC5879472

[B117] Sokoloff L. (1999). Energetics of functional activation in neural tissues. *Neurochem. Res.* 24 321–329. 10.1023/A:1022534709672 9972882

[B118] Song X. Long D. (2020). Nrf2 and Ferroptosis: A New Research Direction for Neurodegenerative Diseases. *Front. Neurosci.* 14:1–15. 10.3389/fnins.2020.00267 32372896 PMC7186402

[B119] Sripetchwandee J. Wongjaikam S. Krintratun W. Chattipakorn N. Chattipakorn S. C. (2016). A combination of an iron chelator with an antioxidant effectively diminishes the dendritic loss, tau-hyperphosphorylation, amyloids-β accumulation and brain mitochondrial dynamic disruption in rats with chronic iron-overload. *Neuroscience* 332 191–202. 10.1016/j.neuroscience.2016.07.003 27403880

[B120] Ståhl P. L. Salmén F. Vickovic S. Lundmark A. Navarro J. F. Magnusson J. (2016). Visualization and analysis of gene expression in tissue sections by spatial transcriptomics. *Science* 353 78–82. 10.1126/science.aaf2403 27365449

[B121] Stockwell B. R. Friedmann Angeli J. P. Bayir H. Bush A. I. Conrad M. Dixon S. J. (2017). Ferroptosis: A Regulated Cell Death Nexus Linking Metabolism, Redox Biology, and Disease. *Cell* 171 273–285. 10.1016/j.cell.2017.09.021 28985560 PMC5685180

[B122] Strozyk D. Launer L. J. Adlard P. A. Cherny R. A. Tsatsanis A. Volitakis I. (2009). Zinc and copper modulate Alzheimer Aβ levels in human cerebrospinal fluid. *Neurobiol. Aging* 30 1069–1077. 10.1016/j.neurobiolaging.2007.10.012 18068270 PMC2709821

[B123] Sultana R. Perluigi M. Butterfield D. A. (2013). Lipid peroxidation triggers neurodegeneration: A redox proteomics view into the Alzheimer disease brain. *Free Radic. Biol. Med.* 62 157–169. 10.1016/j.freeradbiomed.2012.09.027 23044265 PMC3573239

[B124] Taghizadeh M. Tamtaji O. R. Dadgostar E. Daneshvar Kakhaki R. Bahmani F. Abolhassani J. (2017). The effects of omega-3 fatty acids and vitamin E co-supplementation on clinical and metabolic status in patients with Parkinson’s disease: A randomized, double-blind, placebo-controlled trial. *Neurochem. Int.* 108 183–189. 10.1016/j.neuint.2017.03.014 28342967

[B125] Tanaka H. Homma H. Fujita K. Kondo K. Yamada S. Jin X. (2020). YAP-dependent necrosis occurs in early stages of Alzheimer’s disease and regulates mouse model pathology. *Nat. Commun.* 11:507. 10.1038/s41467-020-14353-6 31980612 PMC6981281

[B126] Trombetta-Lima M. Sabogal-Guáqueta A. M. Dolga A. M. (2021). Mitochondrial dysfunction in neurodegenerative diseases: A focus on iPSC-derived neuronal models. *Cell Calcium* 94:102362. 10.1016/j.ceca.2021.102362 33540322

[B127] Wan Y. W. Al-Ouran R. Mangleburg C. G. Perumal T. M. Lee T. V. Allison K. (2020). Meta-Analysis of the Alzheimer’s Disease Human Brain Transcriptome and Functional Dissection in Mouse Models. *Cell Rep.* 32:107908. 10.1016/j.celrep.2020.107908 32668255 PMC7428328

[B128] Wang C. Y. Wang Z. Y. Xie J. W. Wang T. Wang X. Xu Y. (2016). Dl-3-n-butylphthalide-induced upregulation of antioxidant defense is involved in the enhancement of cross talk between CREB and Nrf2 in an Alzheimer’s disease mouse model. *Neurobiol. Aging* 38 32–46. 10.1016/j.neurobiolaging.2015.10.024 26827641

[B129] Wang J. Xiong S. Xie C. Markesbery W. R. Lovell M. A. (2005). Increased oxidative damage in nuclear and mitochondrial DNA in Alzheimer’s disease. *J. Neurochem.* 93 953–962. 10.1111/j.1471-4159.2005.03053.x 15857398

[B130] Wang L. Liu Y. Du T. Yang H. Lei L. Guo M. (2020). ATF3 promotes erastin-induced ferroptosis by suppressing system Xc–. *Cell Death Different.* 27 662–675. 10.1038/s41418-019-0380-z 31273299 PMC7206049

[B131] Wang Y. Liu Z. Lin T. M. Chanana S. Xiong M. P. (2018). Nanogel-DFO conjugates as a model to investigate pharmacokinetics, biodistribution, and iron chelation in vivo. *Int. J. Pharmaceut.* 538 79–86. 10.1016/j.ijpharm.2018.01.004 29341909 PMC5845769

[B132] Ward R. J. Zucca F. A. Duyn J. H. Crichton R. R. Zecca L. (2014). The role of iron in brain ageing and neurodegenerative disorders. *Lancet Neurol.* 13 1045–1060. 10.1016/S1474-4422(14)70117-6 25231526 PMC5672917

[B133] Weiland A. Wang Y. Wu W. Lan X. Han X. Li Q. (2019). Ferroptosis and Its Role in Diverse Brain Diseases. *Mol. Neurobiol.* 56 4880–4893. 10.1007/s12035-018-1403-3 30406908 PMC6506411

[B134] Xu J. Jia Z. Knutson M. D. Leeuwenburgh C. (2012). Impaired iron status in aging research. *Int. J. Mol. Sci.* 13 2368–2386. 10.3390/ijms13022368 22408459 PMC3292028

[B135] Yamamoto A. Shin R. W. Hasegawa K. Naiki H. Sato H. Yoshimasu F. (2002). Iron (III) induces aggregation of hyperphosphorylated τ and its reduction to iron (II) reverses the aggregation: Implications in the formation of neurofibrillary tangles of Alzheimer’s disease. *J. Neurochem.* 82 1137–1147. 10.1046/j.1471-4159.2002.01061.x 12358761

[B136] Yan N. Zhang J. J. (2020). Iron Metabolism, Ferroptosis, and the Links With Alzheimer’s Disease. *Front. Neurosci.* 13:1443. 10.3389/fnins.2019.01443 32063824 PMC7000453

[B137] Yang W. S. Kim K. J. Gaschler M. M. Patel M. Shchepinov M. S. Stockwell B. R. (2016). Peroxidation of polyunsaturated fatty acids by lipoxygenases drives ferroptosis. *Proc. Natl. Acad. Sci. U S A.* 113 E4966–E4975. 10.1073/pnas.1603244113 27506793 PMC5003261

[B138] Yiannopoulou K. G. Anastasiou A. I. Zachariou V. Pelidou S. H. (2019). Reasons for failed trials of disease-modifying treatments for alzheimer disease and their contribution in recent research. *Biomedicines* 7:97. 10.3390/biomedicines7040097 31835422 PMC6966425

[B139] Yoo M. H. Gu X. Xu X. M. Kim J. Y. Carlson B. A. Patterson A. D. (2010). Delineating the role of glutathione peroxidase 4 in protecting cells against lipid hydroperoxide damage and in alzheimer’s disease. *Antioxid. Redox Signal.* 12 819–827. 10.1089/ars.2009.2891 19769463 PMC2861544

[B140] Yu H. Yang C. Jian L. Guo S. Chen R. Li K. (2019). Sulfasalazine-induced ferroptosis in breast cancer cells is reduced by the inhibitory effect of estrogen receptor on the transferrin receptor. *Oncol. Rep.* 42 826–838. 10.3892/or.2019.7189 31173262

[B141] Zhang F. Tao Y. Zhang Z. Guo X. An P. Shen Y. (2012). Metalloreductase steap3 coordinates the regulation of iron homeostasis and inflammatory responses. *Haematologica* 97 1826–1835. 10.3324/haematol.2012.063974 22689674 PMC3590089

[B142] Zhang Y. H. Wang D. W. Xu S. F. Zhang S. Fan Y. G. Yang Y. Y. (2018). α-Lipoic acid improves abnormal behavior by mitigation of oxidative stress, inflammation, ferroptosis, and tauopathy in P301S Tau transgenic mice. *Redox Biol.* 14 535–548. 10.1016/j.redox.2017.11.001 29126071 PMC5684493

[B143] Zhang Z. H. Wu Q. Y. Zheng R. Chen C. Chen Y. Liu Q. (2017). Selenomethionine mitigates cognitive decline by targeting both tau hyperphosphorylation and autophagic clearance in an Alzheimer’s disease mouse model. *J. Neurosci.* 37 2449–2462. 10.1523/JNEUROSCI.3229-16.2017 28137967 PMC6596838

[B144] Zhao R. Z. Jiang S. Zhang L. Yu Z. B. (2019). Mitochondrial electron transport chain, ROS generation and uncoupling (Review). *Int. J. Mol. Med.* 44 3–15. 10.3892/ijmm.2019.4188 31115493 PMC6559295

[B145] Zhao Y. Zhao B. (2013). Oxidative stress and the pathogenesis of alzheimer’s disease. *Oxidat. Med. Cell. Longev.* 14 450–464. 10.1155/2013/316523 23983897 PMC3745981

[B146] Zhou R. P. Chen Y. Wei X. Yu B. Xiong Z. G. Lu C. (2020). Novel insights into ferroptosis: Implications for age-related diseases. *Theranostics* 10 11976–11997. 10.7150/thno.50663 33204324 PMC7667696

[B147] Zorov D. B. Juhaszova M. Sollott S. J. (2014). Mitochondrial reactive oxygen species (ROS) and ROS-induced ROS release. *Physiol. Rev.* 94 909–950. 10.1152/physrev.00026.2013 24987008 PMC4101632

[B148] Zou Y. Palte M. J. Deik A. A. Li H. Eaton J. K. Wang W. (2019). A GPX4-dependent cancer cell state underlies the clear-cell morphology and confers sensitivity to ferroptosis. *Nat. Commun.* 10:1617. 10.1038/s41467-019-09277-9 30962421 PMC6453886

