# Corrigendum: The Potential of Ferroptosis-Targeting Therapies for Alzheimer's Disease: From Mechanism to Transcriptomic Analysis

**DOI:** 10.3389/fnagi.2022.863205

**Published:** 2022-03-08

**Authors:** Nad'a Majerníková, Wilfred F. A. den Dunnen, Amalia M. Dolga

**Affiliations:** ^1^Research School of Behavioural and Cognitive Neuroscience, University of Groningen, Groningen, Netherlands; ^2^Department of Pathology and Medical Biology, University Medical Centre Groningen, University of Groningen, Groningen, Netherlands; ^3^Department of Molecular Pharmacology, Groningen Research Institute of Pharmacy, University of Groningen, Groningen, Netherlands; ^4^Research Institute Brain and Cognition, Molecular Neuroscience and Aging Research (MOLAR), University Medical Centre Groningen, Groningen, Netherlands

**Keywords:** neurodegeneration, iron dysregulation, glutathione, lipid peroxidation, amyloid β

In the original article, there was a mistake in Table 2 as published. In the paper by Gerrits et al. (2021) microglia and astrocytes were analyzed separately (i.e., subcluster 0 of astrocytes is not the same as subcluster 0 from microglia). The corrected Table 2 appears below.

**Table 2:** Log-fold change of ferroptosis-related DEGs in glia cells in AD.

		**Microglia**																
**Subgroups**	**Clusters/**																	
	**genes**																	
		***ACSL1***	***ACSL4***	***ALOX15***	***ATG7***	***FTH1***	***FTL***	***GCH1***	***GCLC***	***HMOX1***	***NCOA4***	***SAT1***	***SLC11A2***	***SLC40A1***	***SLC7A11***	***STEAP3***	***TFRC***	***TP53***
Homeostatic	0																	
	1																	
	5																	
Amyloid beta-related	7																	
	9																	
	10																	
Tau pathology-related	2																	
	3																	
	6																	
Other	8																	
	11																	
	4																	
	12																	

*
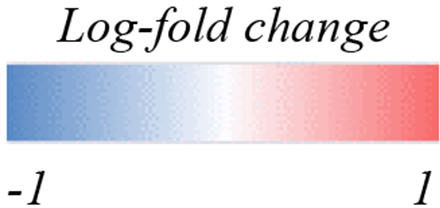
*

In the original article, there was a mistake in the legend for Table 2 as published. The legend was changed because the content of the Table 2 revised, and we used Log-fold change in the color scale legend. The correct legend appears below.

Data in this table represent the Log-fold change per gene per subcluster. Decreased (blue) and increased (red) expression of ferroptosis-related genes in microglia nuclei isolated from CTR and AD brain tissues. Microglia were clustered into 13 subclusters, that were categorized as follows: 1. homeostatic, 2. Aβ-plaque associated (-AD1) and 3. Tau-associated (AD2), and other subclusters were related to pro-inflammatory responses, cellular stress and proliferation. White space corresponds to unchanged gene expression. ACSL, Long-chain-fatty-acid—CoA ligase; ALOX15, coding for Arachidonate 15-lipoxygenase/15-lipoxygenase-1; ATG, Autophagy related gene; FTH1, Ferritin heavy chain; FTL, Ferritin light chain; GCH1, Guanosine triphosphate cyclohydrolase-1; GCLC, Glutamate-cysteine ligase catalytic subunit; HMOX1, Heme oxygenase 1; NCOA4, Nuclear receptor coactivator 4; SAT1, Spermidine/spermine N1-acetyltransferase; SLC, Solute carrier family; STEAP3, *STEAP3* Metalloreductase, TFRC, Transferrin receptor; TP53, tumor protein 53. The differential expression analysis was performed using a logistic regression from which we included ferroptosis-related genes with an adjusted *p*-value < 0.05. Differential gene expression results were extracted from supplementary table 2 from Gerrits et al. (2021).

Due to the change in Table 2, the text describing it was also revised on page 6, section **Differential Expression of Ferroptosis-related Genes in Alzheimer's Disease**, paragraph 5.

To further investigate how ferroptosis could affect glia cells in AD, we looked at the difference in expression of ferroptosis-related genes in microglia between control and AD brains containing only amyloid-β plaques in the occipital cortex (OC) and both amyloid-β and tau pathology in the occipitotemporal cortex (OTC) (Gerrits et al., 2021). In this study, the differential expression analysis was performed using a logistic regression and adjusted *p*-value below 0.05 was used to determine the significance (Gerrits et al., 2021). Microglia belonging to different subclusters (homeostatic, Aβ-related = AD1 and tau-related = AD2) showed changes in the expression of ferroptosis-related genes between AD and control subjects (Table 2). Microglia affected by Aβ pathology alone, or the combination of Aβ and tau pathology showed more DEGs than cells in the homeostatic subcluster. Microglia in the Aβ-related subcluster showed increase in the expression of ferroptosis-related genes, while microglia in tau pathology-related subcluster showed decrease in the expression of these genes. As the presence of tau pathology in OC is typical for later stages of the diseases, these results could suggest that there seem to be a difference between the expression of ferroptosis-related genes between early and late stages of AD. However, whether glia cells die *via* ferroptotic cell death at later stages of AD should be investigated further.

The authors apologize for this error and state that this does not change the main scientific conclusions of the article in any way. The original article has been updated.

## Publisher's Note

All claims expressed in this article are solely those of the authors and do not necessarily represent those of their affiliated organizations, or those of the publisher, the editors and the reviewers. Any product that may be evaluated in this article, or claim that may be made by its manufacturer, is not guaranteed or endorsed by the publisher.
