# Supplementary material for: The Potential of Ferroptosis-Targeting Therapies for Alzheimer’s Disease: From Mechanism to Transcriptomic Analysis
Source: Front Aging Neurosci. 2021 Dec 20;13:745046. doi: 10.3389/fnagi.2021.745046 (PMC8721139; doi:10.3389/fnagi.2021.745046)
Supplement: Supplementary file 1 [file Table_1.docx]

**Supplementary**

**Table S1** Human ferroptosis-related genes

*Ferroptosis-related genes of which nine are not differentially expressed in AD (highlighted in red). ACSL1, Long-chain-fatty-acid—CoA ligase 1; ACSL3, Long-chain-fatty-acid—CoA ligase 3; ACSL4, Long-chain-fatty-acid—CoA ligase 4; ACSL5, Long-chain-fatty-acid—CoA ligase 5; ACSL6, Long-chain-fatty-acid—CoA ligase 6; AIFM2, Apoptosis-inducing factor mitochondria-associated 2; ALOX15, Arachidonate 15-lipoxygenase/15-lipoxygenase-1; ATG5, Autophagy related 5; ATG7, Autophagy related 7; CP, Ceruloplasmin; CYBB, Cytochrome B-245 Beta chain; DHODH, Dihydroorotate dehydrogenase; FTH1, Ferritin heavy chain; FTL, Ferritin light chain; FTMT, Ferritin mitochondrial;* *GCH1, Guanosine triphosphate cyclohydrolase-1; GCLC, Glutamate-cysteine ligase catalytic subunit; GCLM, Glutamate-cysteine ligase modifier subunit; GPX4, Glutathione peroxidase 4, GSS, Glutathione synthetase; HMOX1, Heme oxygenase 1; LPCAT3, Lysophosphatidylcholine acyltransferase 3; MAP1LC3A, Microtubule associated protein 1 light chain 3 Alpha; MAP1LC3B, Microtubule associated protein 1 light chain 3 Beta; MAP1LC3B2, Microtubule associated protein 3 light chain 2 Beta; MAP1LC3C, Microtubule associated protein 1 light chain 3 gamma; NCOA4, Nuclear receptor coactivator 4; PCBP1, Poly(rC)-binding protein 1; PCBP2, Poly(rC)-binding protein 2; PRNP, Prion protein; SAT1, Spermidine/spermine N1-acetyltransferase 1; SAT2, Spermidine/spermine N1-acetyltransferase 2; SLC11A2 Solute carrier family 11 member 2; SLC39A14, Solute carrier family 39 member 14; SLC39A8, Solute carrier family 39 member 8; SLC3A2, Solute carrier family 3 member 2; SLC40A1, Solute carrier family 40 member 1; SLC7A11, Solute carrier family 7 member 11; STEAP3,* STEAP3*Metalloreductase, TF, Transferrin; TFRC, Transferrin receptor; TP53, tumor protein 53; VDAC2, Voltage-dependent anion channel 2; VDAC3, Voltage-dependent anion channel 3. Ferroptosis-related genes were analysed among AD DEGs based on Mathys et al., 2019, Gerrits et al., 2021 and Wan et al., 2020.*
